# *Atonal homolog 1* Is a Tumor Suppressor Gene

**DOI:** 10.1371/journal.pbio.1000039

**Published:** 2009-02-24

**Authors:** Wouter Bossuyt, Avedis Kazanjian, Natalie De Geest, Sofie Van Kelst, Gert De Hertogh, Karel Geboes, Greg P Boivin, Judith Luciani, Francois Fuks, Marinee Chuah, Thierry VandenDriessche, Peter Marynen, Jan Cools, Noah F Shroyer, Bassem A Hassan

**Affiliations:** 1 Laboratory of Neurogenetics, Department of Molecular and Developmental Genetics, VIB, Leuven, Belgium; 2 Department of Human Genetics, K.U. Leuven School of Medicine, Leuven, Belgium; 3 Doctoral Program in Molecular and Developmental Genetics, K.U. Leuven Group Biomedicine, Leuven, Belgium; 4 Division of Gastroenterology, Hepatology, & Nutrition, Children's Hospital Research Foundation, Cincinnati, Ohio, United States of America; 5 Department of Pathology, Leuven University Hospital, K.U. Leuven, Leuven, Belgium; 6 Department of Pathology and Laboratory Medicine, University of Cincinnati College of Medicine, Cincinnati, Ohio, United States of America; 7 Laboratory of Cancer Epigenetics, Faculty of Medicine, Free University of Brussels (U.L.B.), Brussels, Belgium; 8 The Vesalius Research Center, VIB, Leuven, Belgium; 9 The Vesalius Research Center, K.U. Leuven School of Medicine, Leuven, Belgium; 10 The Human Genome Laboratory, Department of Molecular and Developmental Genetics, VIB, Leuven, Belgium; 11 Division of Developmental Biology, Children's Hospital Research Foundation, Cincinnati, Ohio, United States of America; 12 Department of Pediatrics, University of Cincinnati, Cincinnati, Ohio, United States of America; Western General Hospital, United Kingdom

## Abstract

Colon cancer accounts for more than 10% of all cancer deaths annually. Our genetic evidence from *Drosophila* and previous in vitro studies of mammalian *Atonal homolog 1* (*Atoh1*, also called *Math1* or *Hath1*) suggest an anti-oncogenic function for the Atonal group of proneural basic helix-loop-helix transcription factors. We asked whether mouse *Atoh1* and human *ATOH1* act as tumor suppressor genes in vivo. Genetic knockouts in mouse and molecular analyses in the mouse and in human cancer cell lines support a tumor suppressor function for *ATOH1. ATOH1* antagonizes tumor formation and growth by regulating proliferation and apoptosis, likely via activation of the Jun N-terminal kinase signaling pathway. Furthermore, colorectal cancer and Merkel cell carcinoma patients show genetic and epigenetic *ATOH1* loss-of-function mutations. Our data indicate that *ATOH1* may be an early target for oncogenic mutations in tissues where it instructs cellular differentiation.

## Author Summary

Like most cancers, colon cancer displays a loss of differentiation, and the stronger this property, the more aggressive the cancer. This suggests that the loss of the capacity to differentiate may be a critical and possibly early event during the formation of these tumors. The key gene instructing secretory cell fate differentiation in the epithelium of the colon, namely *Atonal homolog 1* (*ATOH1*), is highly conserved in flies, mice, and humans. We asked whether *ATOH1* could be a pivotal factor in causing colon cancer in mice and humans. Our studies show that colon-specific loss of *ATOH1* in mice is sufficient to trigger colon cancer and that the majority of human colon cancers also have an inactivated *ATOH1*. Reactivating *ATOH1* in cultured human colon cancer cells causes these cells to stop dividing and to commit suicide. Since reactivation of this epigenetically silenced gene can be achieved using small chemical compounds, studying how *ATOH1* acts may offer therapeutic avenues in the future.

## Abstract 3

A master regulator of differentiation in the colon is a tumor suppressor gene for colon cancer.

## Introduction

The Atonal (Ato) proneural transcription factors form a highly conserved group of key developmental regulators in multiple neural and neuroendocrine tissues. The mammalian *Ato* (CG7508) ortholog, *ATOH1* (Ensembl accession number: ENSG00000172238), is essential for cell fate commitment of mechanoreceptive Merkel cells in the skin [1] and the secretory goblet, Paneth, and enteroendocrine cells in the intestine [2,3] in addition to multiple neuronal lineages [4,5]. The functional conservation between the fly and mammalian proteins is underscored by the fact that Drosophila ato can fully rescue the *Atoh1* (Ensembl: ENSMUSG00000073043) null mutant mouse [6].

Genetic analyses in *Drosophila* [7] suggest that *ato* regulates the formation and progression of tumors in fly retina, where it acts as a master regulator of cell fate specification. In mammals, two aggressive human cancers derive from tissues where ATOH1 instructs cell fate commitment, namely Merkel cell carcinoma (MCC) and colorectal cancer (CRC). MCC is a rare, but very aggressive, neuroendocrine cancer of the skin with approximately 40% mortality [8]. CRC is a highly prevalent cancer with high mortality (36%) representing 11% of all cancer deaths annually [9]. Recent studies in colon cancer cell lines suggest that ATOH1 can inhibit tumor cell growth in vitro [10]. If the anti-oncogenic function of Drosophila ato is conserved in its mammalian counterparts, one would predict that the loss and gain of function of *ATOH1* would enhance and suppress tumor formation, respectively, in MCC and CRC models. In addition, the ATOH1 should be subject to loss-of-function mutations in a significant number of human cancer patients.

We tested this prediction in two different mouse models for colon cancer, as well as human MCC and CRC cell lines. In addition, we examined the status of the ATOH1 locus in primary tumor samples from human MCC and CRC patients. Our data show that loss of *ATOH1* strongly enhances the formation and progression of tumors in mice and human cell lines. Conversely, gain of *ATOH1* function strongly inhibits the oncogenic phenotypes in human cell lines. Furthermore, we find genetic and epigenetic loss-of-function mutations with very high frequency in primary human tumors derived from *ATOH1*-dependent tissues and provide biochemical insight into how these epigenetic mutations may arise. Finally, we describe a highly conserved anti-oncogenic molecular signaling pathway that links *ATOH1* activity to the stress sensor Jun N-terminal kinase (JNK) pathway mediated via cell type–specific differential co-option of receptor tyrosine kinases (RTKs). Together with the genetic analysis in *Drosophila* [7], these data support a novel, highly conserved tumor suppressor function for the Atonal group of transcription factors.

## Results

### Loss of *Atoh1* Promotes Tumor Formation in Two Colorectal Cancer Mouse Models

In the mouse, Atoh1 is a master regulator of secretory cell fate commitment in the intestinal epithelium [2,11]. To investigate whether Atoh1 plays a role in intestinal tumors, we analyzed the function of the mammalian *ato* homolog, *Atoh1*, in colon tumorigenesis. We assessed the tumor susceptibility of mice with an intestine-specific deletion of *Atoh1* (*Atoh1^Δintestine^*) [3] in two different established mouse models of CRC.

**Figure 1:**
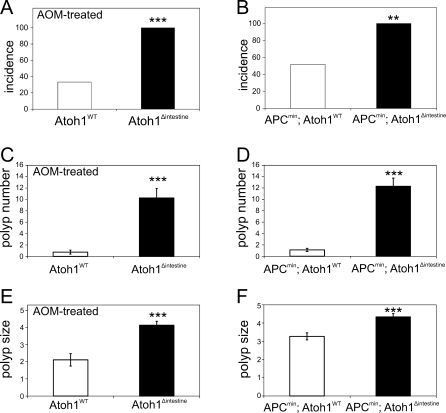
Loss of *Atoh1* Enhances Tumor Formation in the Mouse Colon (A) shows the incidence of polyps in AOM-treated mice. (B) shows the incidence of polyps in the *Atoh1^wt^* and *Atoh1^Δintestine^* mice in an *APC^min^* background. In (A) and (B), the two-tailed Fisher exact test was used. (C) Bar graph shows the average number of macroscopically visible polyps (>1 mm) in the colons of AOM-treated *Atoh1^wt^* (WT; *n* = 9 polyps in 12 mice, white bars) and *Atoh1^Δintestine^* (*n* = 82 polyps in 8 mice, black bars). (D) The average number of polyps in the colons of *APC^min^* (*n* = 27 polyps in 27 mice) and *APC^min^; Atoh1^Δintestine^* (*n* = 123 polyps in 10 mice). (E and F) The average maximum diameter of each polyp is shown as a bar graph for *Atoh1^wt^* or *Atoh1^Δintestine^* mice. (E) Comparison of polyp size from AOM-treated *Atoh1^wt^* and *Atoh1^Δintestine^* mice; and (F) from *APC^min^* and *APC^min^*; *Atoh1^Δintestine^* mice. For (C–F), the two-tailed Student *t-*test was used to measure significance. Error bars indicate the standard error of the mean. Double asterisks (**) indicate *p* <0.01; triple asterisks (***) indicate *p* <0.001.

**Figure 2:**
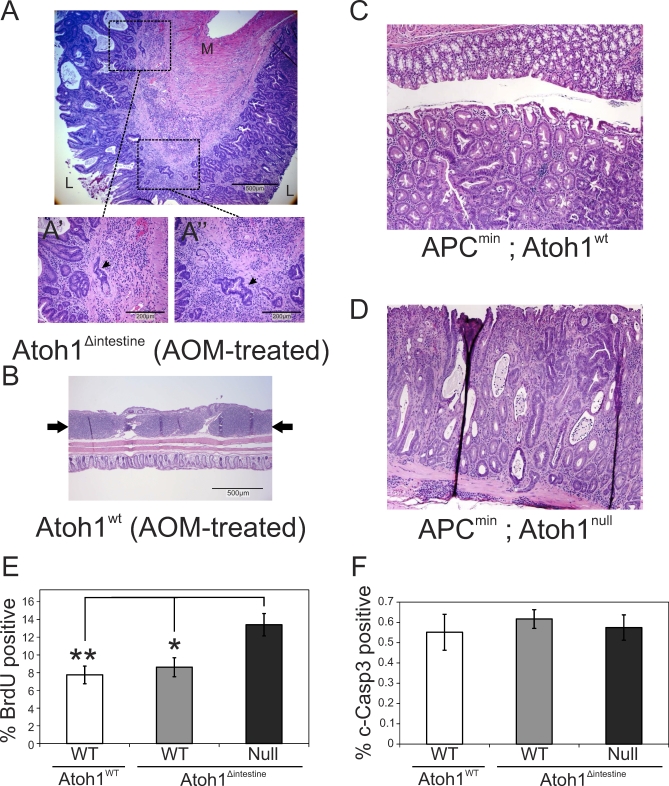
Increased Proliferation Contributes to Tumorigenesis in *Atoh1^Δintestine^* Colon (A) Hematoxylin and eosin staining of a representative adenoma in AOM-treated *Atoh1^Δintestine^* colon (5× magnification). The section is in the rectal region and is characterized by cystic structures with severe crypt hyperplasia with multifocal embedded adenomas and severe lymphocyte infiltration of the submucosa. (L, lumen; M, muscle). (A)′ and (A)′′ show higher magnification (20×) of two areas of the adenoma, indicating the aberrant branched crypt structures that are imbedded in the submucosa. (B) The colons of AOM-treated *Atoh1^wt^* mice showed large GALT (highlighted by arrows) that were macroscopically counted as polyps. Magnification is 5×. Note the relative size of the GALT compared to adjacent normal-appearing crypts. (C) Histological analysis of colon in *APC^min^*. (D) Histological analysis in *APC^min^*; *Atoh1^Δintestine^* mice show highly dysplastic adenomas with cystic structures and lymphocyte infiltration. (E) Proliferation was measured by determining the percentage of BrdU-positive epithelial cells in AOM-treated *Atoh1^wt^* and *Atoh1^Δintestine^* normal-appearing colonic crypts. The white bar represents crypts from *Atoh1^wt^* mice; the gray bar represents nondeleted *Atoh1^wt^* crypts, and the black bar *Atoh1-null* crypts in *Atoh1^Δintestine^* mice. (F) Apoptosis was measured by determining the percentage of cleaved caspase-3 (c-Caspase 3)-positive epithelial cells in AOM-treated *Atoh1^wt^* and *Atoh1^Δintestine^* normal-appearing colonic crypts. Bar shading indicates crypt genotype as in (E). For each graph in (E and F), the two-tailed Student *t-*test was used to measure significance: a single asterisk (*) indicates *p* < 0.05; double asterisks (**) indicate *p* < 0.01. Error bars indicate the standard error of the mean.

We first treated *Atoh1^Δintestine^* mice with azoxymethane (AOM), a chemical carcinogen that preferentially induces colon tumors. We found a significant enhancement in polyp formation in the large intestines of *Atoh1^Δintestine^* compared to wild-type littermate mice, characterized by increased incidence (33% [4/12] vs. 100% [8/8], *p* < 0.005; Figure 1A), multiplicity (0.75 vs. 10.3 polyps/colon, *p* < 0.0008; Figure 1C), and size (2.1 vs. 4.1 mm/polyp, *p* < 0.0004; Figure 1E) when examined under the dissecting microscope. Histological analysis of colons from AOM-treated *Atoh1^Δintestine^* mice confirmed these findings and showed a range of histological phenotypes, from severely hyperplastic mucosa with embedded multifocal adenomas, to large, highly dysplastic adenomas, with one animal having invasive adenocarcinoma (Figure 2A, 2A′, and 2A′′). In contrast, histological examination of AOM-treated *Atoh1^wt^* littermates showed none with adenomatous changes; with all polyp-like structures shown to be gut-associated lymphoid tissue (GALT; Figures 2B and S1). These data support the notion that loss of *Atoh1* can be an initiating event in mammalian cancer formation.

In previous work, we found that ablation of *Atoh1* leads to an increase of proliferation but has no effect on apoptosis [3]. We therefore examined proliferation and apoptosis in the “preneoplastic” normal-appearing epithelium within the colons of AOM-treated *Atoh1^wt^* and *Atoh1^Δintestine^* mice. As in the *Drosophila* model, we find more proliferation of epithelial cells in *Atoh1^Δintestine^* crypts compared to nonrecombined wild-type crypts within the same animals, or compared to crypts from *Atoh1^wt^* littermates (Figures 2E and S2D–S2F) but no obvious difference in apoptosis (Figures 2F and S2G–S2I).

We extended our tumor analysis with an independent genetic mouse model for CRC by crossing *Atoh1^Δintestine^* mice to *APC^min^* mice (APC: ENSMUSG00000005871), in which Wnt signaling is constitutively activated [12,13]. *APC^min^* mice develop spontaneous adenomas primarily in the small intestine, with only occasional polyps in the colon [14]. Interestingly, Wnt signaling is thought to interact negatively with *ato* during sense organ formation in the developing *Drosophila* epithelium [15]. Comparing the large intestines of *APC^min^; Atoh1^wt^* mice to *APC^min^; Atoh1^Δintestine^* littermates at 16 wk, we find significantly more polyps in the colons of double-mutant mice, characterized by increased incidence (52% [14/27] vs. 100% [10/10], *p* <0.007; Figure 1B), multiplicity (1.1 vs. 12.3 polyps/colon, *p* <0.0001; Figure 1D), and size (3.3 vs. 4.3 mm/colon, *p* <0.0002; Figure 1F). Histological examination confirmed these polyps as adenomas in both *APC^min^; Atoh1^wt^* and *APC^min^; Atoh1^Δintestine^* mice, with two polyps in *APC^min^; Atoh1^Δintestine^* mice having progressed to invasive adenocarcinoma (Figure 2C and 2D). The polyps in the *APC^min^; Atoh1^Δintestine^* mice originated from the *Atoh1* mutant crypts as indicated by the absence of goblet cells in the polyps (Figure S3A and S3B).

**Figure 3:**
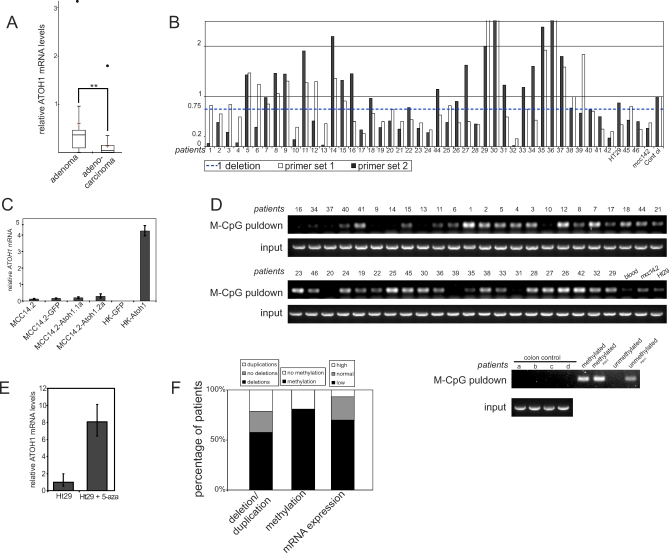
Loss of *ATOH1* Expression and Genomic Deletions in CRC Patient Samples (A) *ATOH1* expression normalized to GADPH and control colon samples: *ATOH1* expression is lower in tumor compared to control samples. Additionally, adenocarcinomas have significantly lower *ATOH1* expression than adenomas. Box plot indicating 25–75 percentiles, central line indicates the median, red cross indicates the mean. Error bars indicate next data point. Outliers are represented as dots. Double asterisks (**) indicate *p* < 0.01 (*t-*test). (B) Deletions in the *ATOH1* locus. Upper dashed line indicates the upper limit of single-deletion detection set at 0.70 of the ratio of *ATOH1* locus to control locus. White and gray bars indicate different primer sets. (C) Endogenous *ATOH1* expression in different MCC14.2-derived cell lines and human keratinocytes (HK) in response to *Atoh1* expression. (D) Detection of methylation at the *ATOH1* locus using pull-down assay of methylated DNA. Bands in “M-CpG pulldown” indicate positive for *ATOH1* methylation. “Input” shows DNA input before the pull-down. Samples were processed blindly. Internal methylated and unmethylated controls are shown in the last four lanes. (E) Inhibition of methyltransferase activity with 5-aza-deoxycytosine for 7 d leads to an increase of *ATOH1* expression in the Ht29 cell line. Error bars indicate the standard deviation. (F) Graph representing observations on the genomic and mRNA levels. First column: percentage of patients showing deletions or duplications (black: deletions, gray: two copies, and white: duplications). Second column: percentage of patients showing methylation using the pull-down of methylated DNA assay (black: methylated, and white: not methylated). Third column: mRNA expression of CRC samples versus control colon (black: low expression, grey: normal expression, and white: high expression).

In summary, thus far, loss-of-function analysis of *Atoh1* in two mouse models of colon tumorigenesis supports a role for *Atoh1* as a key switch in tumor formation and progression. This role appears to be mediated by increased cell proliferation in the absence of *Atoh1*. Importantly, the mouse tumors lack the secretory cell types that depend on *Atoh1* for their formation. Thus, the role of *Atoh1* in tumor formation is likely linked to its function as a master regulator of differentiation.

### Loss of *ATOH1* Expression in Primary Human CRC and MCC Tumors

Given the remarkable evolutionary conservation of *ato*/*Atoh1* function in cancer development, we decided to analyze whether loss of the human ortholog, *ATOH1*, might be selected for during malignant transformation in human cancer. To this end, we investigated the role of *ATOH1* in CRC and MCC. In vitro overexpression experiments in CRC cell lines suggest that *ATOH1* gain of function decreases the population growth potential of CRC cells [10]. Similarly, expression analysis in MCC cell lines suggests an inverse correlation between ATOH1 levels and the population growth of MCC cells (Figure S4A–S4C) [16]. These observations hint at a potential role for ATOH1 in these cancers.

To test this possibility in vivo in primary human tumors, we began by asking whether the expression of *ATOH1* is down-regulated in primary tumor samples from 42 CRC and four MCC patients. As MCC is a very rare cancer, only four samples were available for analysis. Seventy percent of the CRC samples show a significant decrease of *ATOH1* mRNA expression compared to tissue-matched colon samples from normal controls as analyzed by quantitative reverse-transcriptase PCR (RT-qPCR), suggesting that loss of *ATOH1* expression is a highly common feature of CRC oncogenesis (Figures 3F and S4). Furthermore, *ATOH1* mRNA levels are significantly lower in adenocarcinoma samples compared to adenomas (*t-*test: *p* = 0.017), indicating progressive loss of ATOH1 expression levels with increasing tumor severity (Figure 3A). Similarly, in MCC, the two samples with lower *ATOH1* expression levels were derived from the two patients showing metastases (Figure S5).

### CRC and MCC Patients Show Deletions in the *ATOH1* Locus

One mechanism to explain loss of gene expression during oncogenesis is the deletion of the locus, which we tested for using quantitative PCR on genomic DNA in all 46 patient samples, one MCC cell line (MCC14.2), and one CRC cell line (Ht29). At least one deletion in the locus was detected in 57% of the samples (Figure 3B and 3F). A second primer set yielded similar results (49% deletion rate, Figure 3B). One allele was also deleted in both Ht29 and MCC14.2. Comparative genomic hybridization (CGH) array experiments [17] on three samples did not show a deviation of the clones flanking the *ATOH1* locus (Figures S5 and S6), indicating that the deletions observed in patients are likely to be *ATOH1*-specific microdeletions. When only one copy is deleted, complete loss of gene function could be achieved by point mutations in the remaining allele. Sequencing of the *ATOH1* open reading frame of 24 samples, however, did not reveal any mutations in any of the samples (unpublished data). Together with the loss of *ATOH1* mRNA expression in patients, these results suggest that an epigenetic silencing mechanism may be involved—a possibility we tested in further detail.

### The *ATOH1* Locus Is Methylated in CRC and MCC Patients

To address a putative epigenetic transcriptional silencing mechanism, we began by asking whether transcription could, in principle, be initiated from the *ATOH1* locus in cancer cells. Drosophila ato and mouse *Atoh1* are both known to be autoregulatory [18,19]. This provides the opportunity to test whether transcription could be activated from the *ATOH1* locus by ATOH1 itself in normal versus cancer cells. We took advantage of expressing the mouse ortholog in human cells to distinguish the expression of endogenous *ATOH1* from that of *Atoh1*. For the tests in human cancer cell lines, we used the MCC14.2 cell line, which is derived from MCC and has strongly reduced expression of *ATOH1*. Surprisingly, *Atoh1* overexpression failed to activate endogenous human *ATOH1* expression in the MCC14.2 cell line (Figure 3C), despite the virtually complete conservation of the two proteins. This may be due to a key difference between the two proteins, or to a possible disruption of the autoregulatory loop during oncogenesis. To test whether Atoh1 can activate *ATOH1* expression in normal noncancerous skin cells, we transduced normal human primary keratinocytes with an Atoh1 expression vector and tested the expression of endogenous *ATOH1* 40 h after lentiviral transduction. In contrast to the cancer cell lines, endogenous *ATOH1* is up-regulated 38-fold by Atoh1 (Figure 3C). This indicates that the expressability of the *ATOH1* locus is inhibited during oncogenesis.

Genomic database searches show that all known *ATOH1* orthologs, from human to *Drosophila*, reside in a CpG island covering at least the promoter and the transcription start site, suggesting that CpG methylation may be a mechanism of *ATOH1* loss of function. We therefore assayed all patient samples and both cell lines for *ATOH1* methylation using three independent assays: pull-down of methylated DNA, methylation-sensitive restriction digest, and methylation-specific PCR for bisulfite DNA modification. We found that up to 81% of the patients, as well as both cell lines, show methylation of the *ATOH1* locus in both the coding sequence and the promoter sequences directly upstream of the ATG. In contrast, none of the control samples show *ATOH1* methylation (Figures 3D, 3F, and S7A–S7C). This suggests that methylation is likely causal to the transcriptional silencing of the locus during oncogenesis as this is accompanied by lower *ATOH1* expression. In a few cases, however, the correlation was not observed. In two cases (samples 27 and 30), this may be due to the presence of a putative duplication, which is yet to be methylated. Alternatively, but not exclusively, it may be due to heterogeneity of the degree of methylation in different cells comprising the tumor samples analyzed. To provide evidence that methylation silences the *ATOH1* locus, we inhibited DNA methyltransferases (Dnmts) with 5-azadeoxycytidine in the Ht29 cell line. This results in an approximately 8-fold increase in *ATOH1* expression (Figure 3E). Thus, the *ATOH1* locus is methylated in cancer patients and derived cell lines, and this methylation can be reversed, resulting in the transcriptional reactivation of the locus. The autoregulatory function of *ATOH1* combined with the methylation of its regulatory sequences might hint to the involvement of *ATOH1* in the silencing of its own promoter. To gain some insight into whether this may indeed be the case, we asked whether Atoh1 can physically interact with Dnmt proteins. We tested the binding of Atoh1 to Dnmt1 (ENSG00000130816), Dnmt3a (ENSG00000119772), and Dnmt3b (ENSG00000088305) using GST pull-down assays. We find that Atoh1 binds to all three Dnmts. This is further supported by the ability of GST-Atoh1 to pull down DNA methyltransferase activity from nuclear extracts, similar to the positive control (EED: embryonic ectoderm development, ENSG00000074266) [20]. Focusing on Dnmt1, the maintenance DNA methyltransferase, we confirmed this observation using coimmunoprecipitation experiments in 293T cells transfected with HA-tagged Atoh1. Lastly, mapping experiments identified several regions of Dnmt1 as mediating the association with Atoh1 (Figure S8A–S8D).

In summary, deletions and epigenetic silencing via methylation combine to cause loss-of-function mutations of the human *ATOH1* locus in primary human cancers. Together with loss-of-function evidence in five independent cancer models in human cells, *Drosophila*, and mouse, these data strongly support a role for the Ato/ATOH1 transcription factors as key modulators of oncogenic transformation.

### ATOH1 Functions via JNK-Dependent Inhibition of the Cell Cycle and Induction of Apoptosis

**Figure 4:**
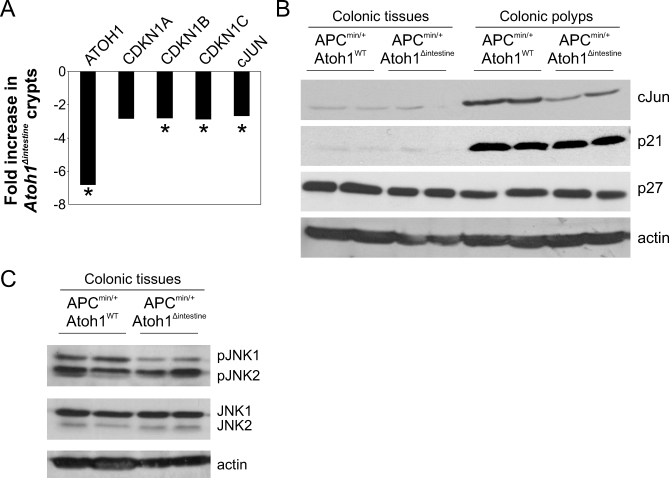
JNK Pathway Influenced by Atoh1 (A) Gene expression analysis in colon crypts from *Atoh1^Δintestine^* and *Atoh1^wt^* mice. Quantitative RT-PCR was used to assess gene expression in isolated colon crypts. The ratio of gene expression is shown, in which negative numbers indicate reduced expression in *Atoh1^Δintestine^* crypts. *t*-tests determined *p*-values shown for each gene. (B) Western analysis of p27^kip^, p21^waf1^, and c-JUN in *APC^min^*; *Atoh1^wt^*, and *APC^min^*; *Atoh1^Δintestine^* colonic tissues and polyps. Representative colonic tissue and polyp lysates of *APC^min^*; *Atoh1^wt^*, and *APC^min^*; *Atoh1^Δintestine^* were used for western analysis of p27^kip^, p21^waf1^, and c-JUN. Actin was used a loading control. p21^waf1^ was significantly up-regulated in polyps compared to nonneoplastic colon tissue. c-Jun protein levels were significantly reduced in colonic polyps upon *Atoh1* loss. p27 is down-regulated in preneoplastic colonic tissue upon loss of *Atoh1*. Quantifications are shown in Figure S12A–S12C. (C) Representative tissues were used for Western analysis of phosphorylated JNK1 and JNK2. Total JNK1 and JNK2 are shown as control. Actin loading control is shown below. pJNK1, but not pJNK2, was significantly reduced in colon tissue from *Atoh1^Δintestine^* compared to *Atoh1^wt^* mice. Quantifications are shown in Figure S12D1–S12D2′.

To better understand the role that ATOH1 plays in cancer, we sought to determine the molecular mechanism by which it acts to suppress the formation and progression of tumors. Gain- and loss-of-function analyses point to *Atoh1*-dependent regulation of proliferation in mouse colon tumors (Figures 2E and S3D–S3F). Analysis of the role of Drosophila ato in fly retinal tumors [7] shows that this function is mediated by the JNK signaling pathway. We hypothesized that the Ato-JNK-p21 pathway may mediate the tumorigenic phenotype in our mouse models of CRC as well. If loss of *ATOH1* were indeed an initiating event in tumor formation, we reasoned that the effects of Atoh1 function would have to be detectable in preneoplastic tissue as this is where oncogenesis begins. We therefore first examined the expression levels of *Atoh1*, *Cdkn1a* (p21, ENSG00000124762), *Cdkn1b* (p27, ENSG00000111276), and *Cdkn1c* (p57, ENSG00000129757) and the JNK target *cJun* (ENSG00000177606) in colon crypts isolated from *Atoh1^wt^* and *Atoh1^Δintestine^* mice (Figure 4A). We observe a 6.8-fold reduction in *Atoh1* expression and an approximately 2.8-fold reduction in expression of all three *Cdkn1* isoforms in colon crypts from *Atoh1^Δintestine^* mice. We also observe a similar reduction in *cJun* mRNA levels. We further examined the Ato-JNK-Cdkn1 pathway by immunoblotting proteins from colon polyps and normal-appearing colon tissue from *APC^min/+^*; *Atoh1^wt^* and *APC^min/+^*; *Atoh1^Δintestine^* mice. Colon polyps show higher levels of cJun and p21^waf^ protein compared to normal colonic tissue. Importantly, however, we find a reduction in cJun levels in *APC^min/+^*; *Atoh1^Δintestine^* polyps compared to *APC^min/+^*; *Atoh1^wt^* polyps, consistent with the mRNA analysis showing less *cJun* (Figure 4B). Importantly, we find a reduction in p27 protein and a trend toward less p21 in *Atoh1*-mutant colon tissues and polyps, consistent with our analysis of mRNA levels (Figure 4B). We also find a specific reduction in pJNK1 levels (Figure 4C) in *APC^min/+^*; *Atoh1^wt^* versus *APC^min/+^*; *Atoh1^Δintestine^* tissues, in agreement with earlier reports that JNK1 (MAPK8: ENSG00000107643) may have anti-oncogenic activity in the mouse intestine [21], whereas JNK2 (MAPK9: ENSG00000050748) deficiency enhances tumorigenesis in other epithelia [22]. Finally, we examined the pattern of JNK activation in the colon of *Atoh1^wt^* and *Atoh1^Δintestine^* mice, and observe identical numbers of pJNK1/2-positive cells in Atoh1-null compared to control crypts (Figure S9). Together with western blot analysis demonstrating selective reduction in pJNK1, but not pJNK2, our data suggest that the level of pJNK1 in individual crypt cells is decreased upon loss of Atoh1. These data are consistent with a preneoplastic function of Atoh1 in regulating proliferation, by a JNK-dependent induction of cell cycle inhibitors.

**Figure 5:**
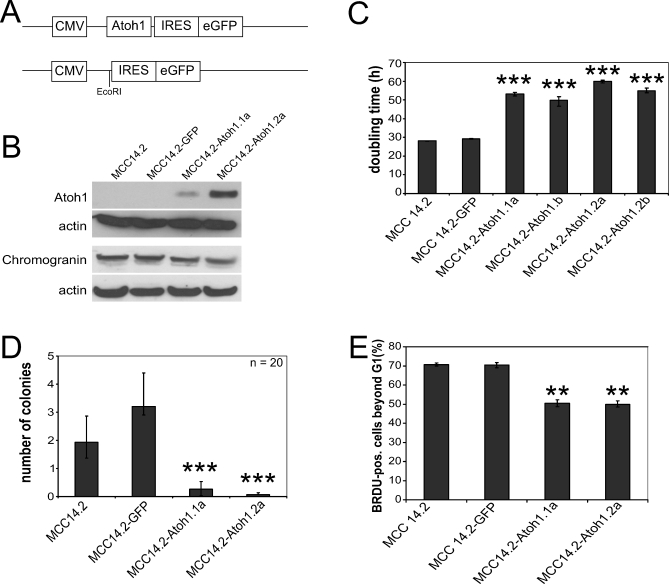
*ATOH1* Suppresses Growth by Interfering with the Cell Cycle (A) Constructs used for creating lentiviral and transfection vectors. (B) Western blot analysis for Atoh1 and chromogranin on untransduced MCC14.2 cells (lane 1), GFP-transduced MCC14.2 cells (lane 2), and two independently derived MCC14.2 cell lines transduced with *Atoh1*-IRES-*eGFP* (lane 3: MCC14.2-Atoh1.1a, and lane 4: MCC14.2-Atoh1.2a). Quantifications in Figure S12E and S12F. (C) Doubling time in hours for MCC14.2 (lane 1), MCC14.2-GFP (lane 2), and four lines independently transduced with *Atoh1*-IRES-*eGFP* (lanes 3–6: MCC14.2-Atoh1.1a, MCC14.2-Atoh1.1b, MCC14.2-Atoh1.2a, and MCC14.2-Atoh1.2b, respectively). (D) Assay for growth in soft agar. Colonies per view with a 10× lens, lane 1: MCC14.2, lane 2: MCC14.2-GFP, lane 3: MCC14.2-Atoh1.1a, and lane 4: MCC14.2-Atoh1.2a. Error bars indicate the 25th and 75th percentiles. The triple asterisks (***) in (C and D) indicate a significant difference from MCC14.2 (*t*-test: *p* < 0.001). (E) Percentage of cells positively labeled for BrdU and past S-phase in a BrdU pulse-chase experiment; double asterisks (**) indicate *p* < 0.01 (*t-*test).

Next we asked whether the molecular mechanism of *ATOH1* function in human cancer is similar to the mouse. We took advantage of several established MCC cell lines [23,24] with either high *ATOH1* expression (MCC1 and MCC6, derived from less aggressive tumors) or low *ATOH1* expression (MCC13, MCC14.2, and MCC26, derived from highly aggressive metastatic tumors; Figure S4A and S4C). We find that the growth rate of cell lines, measured as their doubling time, correlates inversely with levels of *ATOH1* expression (Figure S4B). To determine whether the reduction in ATOH1 levels might be causal to decreased doubling time, we restored ATOH1 function by creating stable cell lines expressing *Atoh1* using lentiviral vectors (Figure 5A and 5B). Stable *Atoh1*-expressing cell lines (MCC14.2-Atoh1.1a, MCC14.2-Atoh1.1b, MCC14.2-Atoh1.2a, and MCC14.2-Atoh1.2b) have a significantly slower population doubling time compared to control cell lines (MCC14.2 and MCC14.2-GFP; *p* <0.0001; Figure 5C). To assess whether this increase in doubling time reflects decreased malignancy, we tested these cell lines for growth in soft agar. Cell lines with high levels of *Atoh1* expression display a marked decrease in growth in soft agar compared to control cell lines (Figure 5D, *p* < 0.001). The change in population doubling time could be due to a slower cell cycle, an increased apoptotic rate, or both. Although the distribution of cells in the cell cycle appears unaltered (Figure S10A), the speed of the cell cycle is 25% lower in *Atoh1*-expressing cells, as assayed by BrdU pulse-chase experiments (Figure 5E, *p* <0.01).

**Figure 6:**
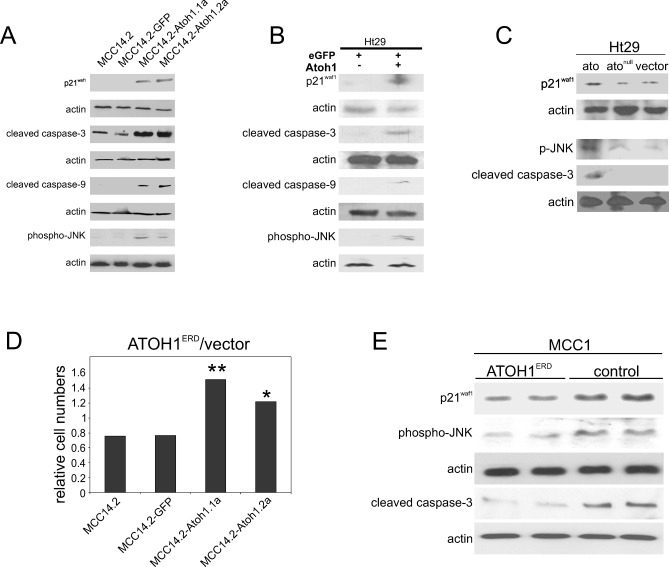
ATOH1 Leads to Activation of Apoptosis and Expression of p21^waf1^ (A) Western blot analysis for p21^waf1^, cleaved caspase-3, cleaved caspase-9, and phosphorylated JNK of lysates of MCC14.2 cells, MCC14.2-GFP, and two MCC14.2 cell lines transduced with *Atoh1*-IRES-*eGFP* (MCC14.2-Atoh1.1a and MCC14.2-Atoh1.2a). The corresponding actin loading controls are shown under each blot. Quantifications are shown in Figure S12G–S12J. (B) Western blot analysis for p21^waf1^, cleaved caspase-3, cleaved caspase-9, and p-JNK of lysates of Ht29 cell line transfected with pCLIG-*eGFP* (left lane) or pCLIG-*Atoh1*-IRES-*eGFP* (right lane); actin loading controls are shown under the respective blots. Quantifications are shown in Figure S12K–S12N. (C) The molecular changes are specific to functional *ato*: western blot of lysates of Ht29 cells transfected with CMV-*ato*, CMV-*ato^1^*, or empty vector. Actin loading controls are shown below the respective blots. Quantifications are shown in Figure S12O–S12Q. (D) Graph expressing ratio of cell numbers of MCC14.2-derived cell lines transfected with pMSCV-*ATOH1^ERD^*-IRES-e*GFP* versus cell number of cell lines transfected with pMSCV-IRES-*eGFP* (vector). Statistical analysis was done using *t-*test under different conditions compared to MCC14.2. Single asterisk (*) indicates *p* < 0.05; double asterisks (**) indicate *p* < 0.01. (E) MCC1 cells transfected with dominant-negative *ATOH1^ERD^* fusion (lanes 1 and 2) and with empty vector control (lanes 3 and 4). Western blot analysis for p21^waf1^, phosphorylated JNK, and cleaved caspase-3 on lysates of MCC1 cell line. The actin loading control of each blot is shown below. Quantifications are shown in Figure S12R–S12T.

In addition to slower proliferation, we find a specific and strong increase in apoptotic cell death, as measured by annexin-V and cleaved caspase-3 (ENST00000308394), in MCC (MCC14.2 series) and CRC (Ht29) cell lines transduced or transfected with Atoh1 (Figures 6A, 6B, and S10B). This increase in cell death is mediated by the intrinsic apoptosis pathway, as suggested by enhanced caspase-9 (ENST00000333868) cleavage (Figure 6A and 6B).

Data from mouse models indicate an involvement of JNK-mediated regulation of cell proliferation for the tumor suppressor effect of Atoh1. To assess whether the same mechanisms are operating in human cancer, we tested the expression levels of the caspase-3 and caspase-9, p21^waf1^, and p-JNK in MCC and CRC cells lines. We note a clear and specific up-regulation of p21^waf1^ and p-JNK levels upon *Atoh1* expression (Figures 6A, 6B, and S10C–S10H). Similarly, transfection of wild-type *Drosophila* Ato results in the up-regulation of cleaved caspase-3, p21^waf1^, and p-JNK (Figure 6C), in contrast to a null-mutant form of the protein that completely fails to bind DNA [25], indicating the conservation and specificity of the Ato/Atoh1 effect.

Next, we tested the expression of these proteins in a loss-of-function setting. We generated and expressed the transcriptional repressor form of ATOH1 by fusing it to the engrailed repressor domain (ATOH1^ERD^) [26], which specifically inhibits the Atoh1-induced effects on MCC cell lines (Figure 6D). In the MCC1 cell line, which shows higher endogenous *ATOH1* expression compared to MCC14.2 (Figure S4C), expression of ATOH1^ERD^ leads to a down-regulation of p21^waf1^ and p-JNK as well as inhibition of caspase-3 cleavage (Figure 6E).

In summary, gain- and loss-of-function studies in mouse colon cancer models and human cancer cells support a conserved antitumor function for *ATOH1* mediated by JNK and p21.

### *ATOH1* Activates RTK Expression in Human Cancer Cells

How does *ATOH1* expression lead to JNK activation in cancer cells? *ATOH1* and its orthologs are known to exert their developmental functions by modulating Notch; Atonal orthologs inhibit Notch signaling to induce differentiation during development, whereas Notch inhibits ato expression by its target gene *Hes1* (ENSG00000114315) [27–29]. Notch signaling has also been described to be upstream of JNK [30]. However, three lines of evidence suggest that the *Atoh1*-mediated effects are not Notch-dependent. First, *Atoh1* expression levels do not influence expression of *HES1*, a Notch signaling target gene (Figure S11A). Second, we do not detect cleaved intracellular Notch in the MCC14.2 cell line, which expresses low levels of *ATOH1* (Figure S11B). Finally, blocking of Notch activation by selective inhibition of γ-secretase [31] has no effect on the growth of MCC cells (Figure S11C). Other known upstream activators of JNK are RTKs [32]. As Atoh1 is a transcription factor, we checked mRNA expression of all 90 human RTKs upon *Atoh1* expression in both the MCC14.2 and the Ht29 cell line compared to control cells. We observed a significant and specific *Atoh1*-dependent up-regulation of Neurotrophic tyrosine kinase receptor type 1 (NTRK1: ENSG00000198400), a hallmark for differentiation in Merkel cells [33,34] in MCC14.2, and of FGF receptors in Ht29 (Figure S11D). The increase in NTRK1 expression in MCC14.2 seen on the mRNA level was confirmed using RT-qPCR (Figure S10E). We also observed elevated NTRK1 levels in the endogenously *ATOH1*-expressing MCC1 cells (Figure S10E). This was accompanied by higher protein levels of both the NTRK1 receptor and one of its ligands, Neurotrophin-3 (NT3: ENSG00000185652; Figure S10E–S10H), also a marker for Merkel cell differentiation. Therefore, Ato gain of function results in tumor type–specific elevation of RTK levels.

### ATOH1 Functions by RTK-Mediated JNK Activation

**Figure 7:**
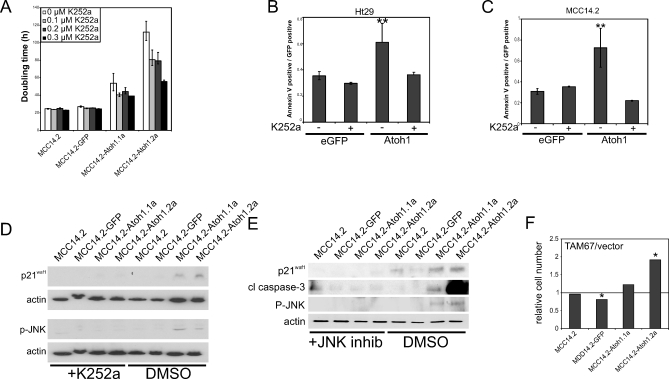
Molecular Mechanism for ATOH1 Function (A–D) show that ATOH1′s anti-oncogenic function acts through receptor tyrosine kinases. (A) Doubling times in hours of MCC14.2 and MCC14.2 transduced with *eGFP* (lane 2) or *Atoh1*-IRES-*eGFP* (lanes 3 and 4) with increasing concentrations of K252a. (B) AnnexinV-positive signals normalized to transfected MCC14.2 cells (GFP). Cells transduced with *eGFP* (lanes 1 and 2) and *Atoh1*-IRES-*eGFP* (lanes 3 and 4) with 0.33 μM K252a (lanes 2 and 4) or DMSO as a control (lanes 1 and 3). The double asterisks (**) indicate significant difference from GFP transfected without K252a (*t*-test: *p* < 0.05). (C) AnnexinV-positive signals normalized to transfected Ht29 cells (GFP). Cells transfected with *eGFP* (lanes 1 and 2) and *Atoh1*-IRES-*eGFP* (lanes 3 and 4) with 0.33 μM K252a (lanes 2 and 4) or DMSO as control (lanes 1 and 3). The double asterisks (**) indicate significant difference from GFP transfected without K252a (*t-*test: *p* < 0.05). (D) Inhibition of RTKs inhibits p21^waf1^ expression and JNK phosphorylation. Western blot analysis for p21^waf1^ and pJNK of MCC14.2 (lanes 1 and 5), MCC14.2 transduced with *eGFP* (lanes 2 and 6), and two MCC14.2-derived cell lines transduced with *Atoh1*-IRES-*eGFP* (lanes 3, 4, 7, and 8) with 0.3 μM K252a (lanes 1–4) and with DMSO as a control (lanes 5–8). Actin loading controls are shown below each blot. Quantifications are shown in Figure S12U and S12V. (E) JNK regulates apoptosis and p21^waf1^ expression. Western blot analysis for p21^waf1^ and cleaved caspase-3 of MCC14.2 (lanes 1 and 5), MCC14.2 transduced with *eGFP* (lanes 2 and 6), and two MCC14.2-derived cell lines transduced with *Atoh1*-IRES-*eGFP* (lanes 3, 4, 7, and 8) with 1 μM JNK inhibitor (lanes 1–4) and with DMSO as a control (lanes 5–8). Actin loading control is shown below the blots. Phospho-JNK (P-JNK) blot shows the effect of the JNK inhibitor on JNK phosphorylation status Quantifications are shown in Figure S12W–S12Y. (F) Relative cell numbers (the number of Tam67-transfected cells divided by the number of mock-transfected cells) for MCC14.2 and MCC14.2 transduced with *eGFP* (lane 2) or *Atoh1*-IRES-*eGFP* (lanes 3 and 4) with dominant-negative c-jun (TAM67).

To test whether the RTKs may be functionally linked with ATOH1, we incubated the various MCC cell lines with K252a, a narrow-specificity RTK inhibitor (NTRK, FGFR, PDGFR, and IGFR) [35–37]. This results in a dose-dependent decrease in doubling time of the MCC14.2-derived, *Atoh1*-expressing cell lines, indicating that the *Atoh1*-induced change in doubling time is RTK-dependent (Figure 7A). In addition, the induction of apoptosis by *Atoh1* in the MCC14.2 and the Ht29 cell line is blocked by RTK inhibition (Figure 7B and 7C). These effects are accompanied by RTK inhibitor–dependent decrease in the expression of p21^waf1^ and p-JNK (Figure 7D), suggesting that both p21^waf1^ and p-JNK are downstream effectors of *Atoh1*-induced RTK signaling. Next, we asked whether p-JNK is required for p21^waf1^ up-regulation and apoptosis by treating MCC cells with SAPK Inhibitor II, a specific JNK inhibitor [38]. We observed a decrease in the *Atoh1*-induced expression of p21^waf1^ and cleaved caspase-3 (Figure 7E). Similarly, transfection of dominant-negative *c-jun* (TAM67) [39] leads to a significant increase in cell number compared to mock-transfected cells (*t-*test: *p* = 0.04), specifically in the MCC14.2-Atoh1.2a, which has high Atoh1 expression levels (Figure 7F).

**Figure 8:**
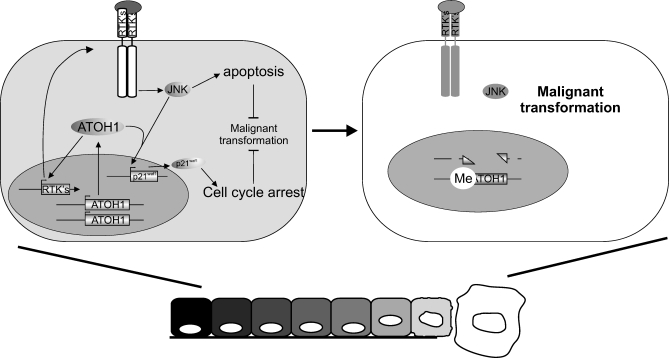
Schematic Representation of the Potential Mechanism of ATOH1′s Function as a Tumor Suppressor In preneoplastic tissue, *Atoh1* keeps malignant transformation in check in a JNK-dependent mechanism by the induction of apoptosis and the inhibition of cell cycle progression. When *Atoh1* is lost due to deletion or methylation, these brakes on oncogenesis fail, and malignant transformation can progress.

In summary, taken together, evidence from mutation analysis in human patients, as well as gain- and loss-of-function analysis in mouse and human cells, support a model (Figure 8) in which ATOH1 modulates JNK activity, possibly via co-option of context-specific RTK signaling, to induce apoptosis and up-regulate p21^waf1^ expression, keeping tumor growth in check. Loss-of-function mutations in *ATOH1* prevent JNK-mediated apoptosis and p21-mediated cell cycle arrest, leading to enhanced tumor progression.

## Discussion

Our data support an evolutionarily conserved tumor suppressor role for *ATOH1* in CRC and MCC. Loss of *ATOH1* promotes tumor formation and progression, and mutations in the *ATOH1* locus are found with relatively high frequency. Given the high deletion and methylation rate of ATOH1 in human tumor samples, loss of *ATOH1* function is likely to be an early event in these tumors. We therefore propose that *ATOH1* acts as a key switch regulating the transformation of pre-oncogenic epithelia to neoplastic and metastatic tumors.

Genetic analysis of the function of Drosophila ato in fly eye tumor suggests that its anti-oncogenic function is linked to its activity as a regulator of cell fate commitment and differentiation [7]. This is similar to what we observe in the different mouse models, where the adenomas and adenocarcinomas do not contain secretory cells (Figure 2) [3]. Interestingly, this also appears to be the case in human cancer, where the majority of human CRCs do not contain differentiated secretory cells. The loss of *ATOH1* in most human CRC patients likely explains this observation. In addition, cell type–specific RTK differentiation genes are up-regulated upon overexpression of *Atoh1* in CRC and MCC cell lines. Importantly, these markers of differentiation are necessary for the anti-oncogenic effect of *ato*/*ATOH1*.

It is tempting to speculate that in other tissues, similar loss of differentiation factors is involved in oncogenesis. In this sense, the loss of differentiation factors has already been implicated in late stages of tumor progression as with GATA-3 (ENSG00000107485) in breast cancer [40]. Although loss of GATA-3 in early tumor leads to an inhibition of tumor formation, loss of GATA-3 in later stages leads to the acquisition of metastatic potential. We, therefore, wonder whether loss of other differentiation factors, perhaps bHLH proteins, might play a role in earlier stages of breast cancer development.

Tumor suppressor genes are defined by the fact that (1) loss-of-function mutations make the cells more prone to malignant transformation, (2) overexpression leads to inhibition of the malignant phenotype, and (3) spontaneous somatic mutations are found in patients with cancer. “Classical tumor suppressor genes,” such as *p53* (ENSG00000141510), are special in the sense that when mutated, the cell is more prone to accumulate additional mutations, and thus actively drive malignant progression as opposed to just “taking away the brakes” [41]. It is notable that *ato/Atoh1* shows most of the hallmarks of a tumor suppressor gene. Because silencing of *ato*/*Atoh1* is not sufficient to drive oncogenesis, we suggest that *ato*/*Atoh1*, and similar genes, are important brakes on malignant transformation. Therefore, the role that differentiation factors might play as key switches in malignant transformation in different tissues is not different from the classical definition of tumor suppressor in a functionally relevant sense.

The RTK and JNK signaling pathways, which are essential for *Atoh1*'s tumor suppressor activity, have been suggested as context-dependent oncogenes or tumor suppressors [42]. Our data indicate that *Atoh1* is important in deciding this context by the up-regulation of cell type–specific RTKs. The activation and co-option of RTK and JNK signaling by Ato/ATOH1 in this context suggests that the status of differentiation of the tumor-initiating cell may be the key determinant of the specific role of various signaling pathways in cancer. This may have important clinical implications: treatment of CRC or MCC patients with RTK or JNK inhibitors might have an adverse effect on tumors where *ATOH1* is still expressed.

In this medical context, our data suggest that screening for *ATOH1* expression, deletion, and methylation may be a useful diagnostic tool for early detection and treatment decision of MCC and CRC. Similarly, treatment of CRC and MCC patients whose tumors show epigenetic silencing of *ATOH1* with DNA methyltransferase inhibitors might prove a powerful avenue for therapy, because it appears to be sufficient to restore *ATOH1* expression and induce cancer cell death. Furthermore, such treatment in combination with Notch inhibitors may enhance re-expression of the ATOH1-driven differentiation program [43,44] and synergistically inhibit cancer growth. Therefore, elucidation of the basic mechanisms of *ato/ATOH1* function, as well as their target genes and interacting proteins, might offer potential avenues for future therapeutic intervention.

## Materials and Methods

### Cloning of ATOH1^ERD^ expression construct.

The *ATOH1* open reading frame was PCR amplified and fused in frame with the engrailed repressor domain after XhoI restriction digest. This fusion product was then blunt ligated in the MSCV-IRES-GFP vector (Clontech), giving rise to the MSCV-*ATOH1^ERD^*-IRES-GFP construct.

### Mouse models and treatments.

*Atoh1^Δintestine^* mice are a conditional deletion of *Atoh1* and are described in more detail elsewhere [3,45]. These mice were generated using the loxP/cre system in which Cre-mediated deletion of *Atoh1* is mosaic and is restricted to the distal ileum and large intestine (80%–90% deletion). *APC^min^* mice were purchased from The Jackson Laboratory and mated with *Atoh1^Δintestine^* mice to generate *APC^min^*; *Atoh1^Δintestine^* mice. Eight-week-old male *Atoh1^wt^* and *Atoh1^Δintestine^* littermates were injected intraperitoneally with AOM (Midwest Research Institute of the National Cancer Institute's chemical carcinogen repository) at 10 mg/kg body weight. AOM was injected weekly for a total of six injections, and the mice were sacrificed 20 wk after the first AOM injection. Two hours prior to sacrifice, mice were injected with 50 mg/kg BrdU. The large intestine was isolated and flushed with PBS and then fixed in 10% buffered formalin at room temperature for 16–24 h. Colons were placed in 70% ethanol before macroscopic analysis of polyps, and then embedded into paraffin blocks. Similarly, 16–19-wk-old *APC^min^* and *APC^min^*; *Atoh1^Δintestine^* colons were isolated and fixed as for the AOM-treated colons. For macroscopic analysis, the position and diameter (millimeters) of each polyp were recorded using a dissecting microscope. The protocol for use of animals was approved by the Cincinnati Children's Hospital Institutional Animal Care and Use Committee.

### Immunohistochemistry and microscopic analysis.

The colons were imbedded in paraffin blocks and sectioned for hematoxylin and eosin staining by the University of Cincinnati pathology core facility. Tumor phenotypes were determined as in Boivin et al. (2003) [46]. Paraffin-embedded colons of AOM-treated mice were sectioned at 5 μm and used for BrdU and cleaved caspase-3 staining. Mouse anti-BrdU antibodies were obtained from the Developmental Studies Hybridoma Bank maintained by the Department of Biological Sciences of the University of Iowa. The sections were de-paraffinized, rehydrated, and antigen retrieval was performed in citric acid buffer (pH = 6) using a microwave. Endogenous peroxidase activity was blocked with hydrogen peroxide/methanol solution, and Avidin/Biotin block was performed according to the manufacturer's recommendations (Vector Laboratories). Endogenous immunoglobulins block and primary and secondary antibody incubations were performed using the M.O.M. kit following the manufacturer's recommendations (Vector Laboratories). Anti-BrdU antibody was incubated for 16 h at 4 °C in a humidified chamber. Color was developed using the DAB peroxidase substrate kit (Vector Laboratories) followed with hematoxylin staining and dehydration of the tissues. Nuclei from well-oriented crypts in *Atoh1^wt^* and *Atoh1^Δintestine^* colons were counted at 40× magnification, followed by counts of BrdU-positive cells. The percent of BrdU-positive *Atoh1^wt^* or *Atoh1-null* cells was determined for each animal (at least 1,000 cells per genotype were counted for each animal). Student two-tailed *t-*test was performed to measure significance. Cleaved caspase-3 staining and counting were performed similarly to BrdU staining and analysis. Polyclonal rabbit anti–cleaved caspase-3 antibodies (1:100) were obtained from Cell Signaling Technology. The Rabbit IgG VECTASTAIN ABC kit was used according to the manufacturer's recommendations for blocking, antibody incubations, and signal amplification (Vector Laboratories).

### Crypt preps.

Crypts from wild-type and *Atoh1^Δintestine^* mice were isolated using a modification of the Evans method [47,48]. Mice were sacrificed and the colons removed and flushed with ice-cold PBS, opened flat, and then placed in cold PBS containing protease and phosphatase inhibitors. After a brief vortexing, the colon was cut into four pieces and placed into a 15-ml tube containing shaking solution (1.5 mM KCL, 96 mM NaCl, 27 mM Na Citrate, 8 mM KH_2_PO_4_, 5.6 mM Na_2_HPO_4_, 15 mM EDTA, and 1 mM dithiothreitol supplemented with protease and phosphatase inhibitors). The tubes were agitated at 4 °C on a vortexer holding the tubes at 180° until the solution appeared cloudy (5–8 min). The colon pieces were transferred into new tubes with shaking buffer and agitated until most of the crypts were released (enrichment was assessed by phase contrast microscopy at different time points). The solution containing the crypts was filtered through a 100-μm cell strainer to isolate the crypts, and sorbitol (2% final concentration) was slowly added and mixed immediately. The solution was centrifuged at 160*g* for 8 min at 4 °C. The supernatant (containing single cells) was removed, and the pellet (containing purified crypts) was snap frozen in liquid nitrogen, to be used for protein and RNA analyses.

### Quantitative reverse-transcriptase polymerase chain reaction.

RNA was purified from colon crypts isolated from wild-type and *Atoh1^Δintestine^* mice using Trizol (Invitrogen) according to the manufacturer's recommendations. Trizol-purified RNA (100 μg) was subjected to DNase digestion and further purification (RNeasy Mini; Qiagen); 2 μg of total RNA was reverse transcribed (Superscript III, Invitrogen), and cDNA equivalent to 100 ng of RNA used for SYBR Green–based real-time PCR using an Mx3005 (Stratagene). For each gene assessed, colon crypt RNA from nine wild-type and eight *Atoh1^Δintestine^* mice was compared using the standard curve method of relative quantification. All gene expression levels were normalized to the expression of GAPDH. *t-*Tests measured significant differences between the average normalized expression levels in wild-type versus *Atoh1^Δintestine^* mice.

### Whole-tissue/polyp preparations.

Cecal and colonic tissues from wild-type and *Atoh1^Δintestine^* mice were homogenized in lysis buffer (1× Phosphate Buffered Saline, 1% NP-40, 0.5% sodium deoxycholate, 0.1% SDS, 0.7 mM EDTA supplemented with protease and phosphatase inhibitors), sonicated, and the lysates used for quantitation and western blots. Similarly, lysates of colonic tissues and polyps from *APC^min^*, and *APC^min^*; *Atoh1^Δintestine^* double-mutant mice were used for whole lysate preparation and western blots. The following antibodies were used for western blot analysis: p21^waf1^ mouse monoclonal antibody (1:500, cat #556431; BD Pharmingen), actin mouse IgM antibodies (1:100, JLA20; Developmental Studies Hybridoma Bank), mouse monoclonal p27^kip1^ antibody (1:250, cat #610241; BD Transduction Laboratories), goat anti-p57 polyclonal antibodies (1:100, cat # sc-1039; Santa Cruz Biotechnology), rabbit polyclonal c-Jun antibodies (1:1000, cat # sc-1694; Santa Cruz Biotechnology), and pJNK antibody (1:1000, cat #559309; EMD-Calbiochem).

### pJNK IHC.

Formalin-fixed tissues from AOM-treated mice were used for phosphorylated-JNK (pJNK) immunohistochemistry. Rabbit polyclonal pJNK antibody was used (1:100, cat #559309; EMD-Calbiochem). The sections were de-paraffinized, hydrated, and the antigen retrieval was performed in citric acid buffer using a microwave. Endogenous peroxidase activity was blocked with hydrogen peroxide/methanol solution, a short remobilization step was included (0.2% Triton X 100 in PBS), and Avidin/Biotin block was performed according to the manufacturer's recommendations (Vector Laboratories). The Rabbit IgG VECTASTAIN ABC kit was used according to the manufacturer's recommendations for blocking and antibody incubations (Vector Laboratories). Primary antibody was incubated at 4 °C for 14–16 h; color was developed using the DAB peroxidase substrate kit (Vector Laboratories) followed by hematoxylin staining and coverslipping. Cells of well-oriented crypts in wild-type and *Atoh1^Δintestine^* colons were counted at 40× magnifications, followed by counts of pJNK-positive cells. The total number of cells and pJNK-positive cell numbers were added for each animal, and the average numbers were compared across crypts and animals. A Student *t-*test was performed to measure significance.

### Cell culture.

MCC cell lines were cultured in RPMI medium supplemented with 15% FCS (Perbio). The Ht29 cell lines (obtained from Deutsche Sammlung von Mikroorganismen und Zellkulturen [DSMZ]) were cultured in McCoy medium supplemented with 10% FCS. Primary human keratinocytes were isolated and pooled from foreskins of three different donors (less than 6 y). Fourth passage cells were used in the experiments. The procedure has been approved by the ethical committee of the University of Leuven. Experiments performed adhered to the Declaration of Helsinki Principles. Keratinocytes were seeded in serum-free and growth factor–containing medium (Keratinocyte-SFM; Invitrogen), which contains several growth factors (5 μg/ml insulin, 74 ng/ml hydrocortisone, 6.7 ng/ml triiodo-l-thyronine, 50 μg/ml bovine pituitary extract, and 5 ng/ml human recombinant EGF). All culture experiments were done at 37 °C in 5% CO_2_.

### Doubling time.

A fixed amount of cells was seeded in standard culture conditions, and the number of cells was counted after 3 to 5 d. The doubling times (*T* ^2X^) were calculated using the formula *T* ^2X^ = LN(2)/((LN(*n*_Δ*t*_)-LN(*n*_0_))/Δ*t*) with Δ*t*, time in culture, *n*_0_, number of seeded cells, and *n*_Δ*t*_ the number of cells after Δ*t*

### Lentiviral vectors.

The lentiviral vector HIV-CMV-Atoh1-IRES-GFP was constructed by cloning a PCR fragment of Atoh1-IRES into the HIV-CMV-GFP vector [49], which was used as control vector. The HIV-CMV-GFP vector was first restricted with XbaI and Age1, and ligated to the Atoh1-IRES fragment, which was spanned between Xba1 and Age1. The Atoh1 and GFP were expressed from a bicistronic vector under the control of the CMV promoter. These lentiviral vectors were produced as described in [50,51]. Stable cell lines were created by transducing the cells in normal medium, supplemented with 8 nM polybrene and a multiplicity of infection between 100 and 150). Four stable cell lines were made with the lentiviral vectors expressing Atoh1 and eGFP from a bicistronic *Atoh1*-IRES-*eGFP* construct (MCC14.2-Atoh1.1a, MCC14.2-Atoh1.1b, MCC14.2-Atoh1.2a, and MCC14.2-Atoh1.2b), and one with eGFP alone (MCC14.2-GFP) as a negative control, under the control of a CMV promoter (Figure 5A). We failed to created stable cell lines from the Ht29. Cells were heavily selected against, and FACS isolation did not yield surviving cells.

### Colony formation in soft agar.

A total of 2,500 cells/ml were resuspended in 0.6% agarose (Invitrogen) in culture medium. A 2-ml layer of 0.35% agarose in culture medium was added on top of the 2-ml 0.6% layer. After 1 wk, 2 ml of 0.35% agarose in culture medium was added. Cells were cultured for 2 wk in standard conditions, and the number of colonies was analyzed under an inverted microscope with a 10× magnification. The experiment was done in triplicate.

### Cell cycle distribution and progression.

Cells were grown up to 40% confluency under standard conditions and were trypsinized. Cells were fixed and permeabilized using 70% ice-cold ethanol for 2 h. Cells were washed in PBS, and cells were stained for 30 min using 0.1% (v/v) Triton X-100 (Sigma) in PBS and 0.2 mg/ml DNase-free Rnase A (Sigma) and 20 μg/ml PI (Sigma). Cells were analyzed on a FACSCalibur cytometer (BectonDickinson). To analyze the cell cycle progression, cells were pulsed for 20 min with 10 μM BrdU (Sigma). Medium was then substituted with normal medium for 6 h. Cells were collected and fixed in 70% ice-cold ethanol. DNA was denatured using 2 M HCl for 20 min. Cells were stained using anti-BrdU and IgG-alexa555 and analyzed in a FACSCalibur (BD Bioscience).

### Apoptosis detection.

Annexin-V staining was performed using Annexin-V-biotin (Roche) and streptavidin-PercP (BD biosciences) as described by the manufacturer. Cells were imaged with the Leica TCS Sp2 confocal microscope, and images were analyzed and quantified using LCS software.

### Western blotting.

Lysates were obtained by scraping cells in medium and centrifuging the medium. The pellet was resuspended in lysis buffer containing PBS with 1 mM EDTA, 1 mM EGTA, 50 mM NaF, 1% TritonX, 5 mM Na_3_VO_4_, 20 μM PAO, and Complete Protease Inhibitor. The crude extract was separated by SDS-PAGE in a 4%–12% NuPAGE novex bis-tris gel (Invitrogen) and electroblotted onto Hybond-ECL membrane (Amersham). Antibodies were diluted in the appropriate concentrations in 5% BSA in TBS-tween20. The antibodies used are beta-actin (clone AC-15; Sigma), anti–cleaved caspase-3 (Asp-175; Cell Signaling Technology), polyclonal antibody to caspase-9(active) (ALEXIS), chromogranin A+B (Abcam), cyclinA1 (Santa Cruz Biotechnology), pJNK (Thr183/Tyr185; BioSource), p27 (BD Bioscience), p21^waf1^ (DCS60; Cell Signaling Technology), PCNA (clone PC10; Sigma), TRK (Santa Cruz Biotechnology), Atoh1 (1/50; Developmental Studies Hybridoma Bank), NT3 (Santa Cruz Biotechnology), and Cleaved Notch (Val1744; Cell Signaling Technology). All western blot analyses are quantified in Figure S11.

### Tyrosine kinase inhibitor, SAPK inhibitor II, and γ-secretase inhibitor.

K252a diluted in DMSO, from VWR, and DMSO as control were used. Final concentrations ranged from 0 μM to 0.5 μM; when no concentration is mentioned, 0.33 μM was used. SAPK inhibitor II, from Calbiochem, was used at a concentration of 10 μM. We used inhibitor X as a γ-secretase inhibitor (Calbiochem). Concentrations used are mentioned in the figure legend.

### RT-PCR and qPCR.

mRNA was amplified using the Superscript II One-Step RT-PCR system with Platinum Taq-100 reactions (Invitrogen) and the *ATOH1* primers CAGCCAGTGCAGGAGGAAAA and GAAAATTCCCCGTCGCTTCT, and the *Hes1* primers GGACATTCTGGAAATGACAGTGAA and AGCGCAGCCGTCATCTG, according to the manufacturer's protocol. Quantitative RT-PCR was performed on a ABI prism 7000 (Applied Biosystems). The primers were designed using Primer Express (Applied Biosystems). The primers used are *ATOH1*: CAGCCAGTGCAGGAGGAAAA and GAAAATTCCCCGTCGCTTCT; *Atoh1*: GCTGTGCAAGCTGAAGGG and TCTTGTCGTTGTTGAAGG. Primers to check copy number of the *ATOH1* locus: *ATOH1* locus set 1: CCCCGGGAGCAATCTTG and GGGACCGAGGCGAAGTT; control locus set 1: TCTGGGACCTGAGCTAATGGA and GGCCATAATTAGGACCATGAAAGA; and *ATOH1* locus set 2: GCCAGTGCAGGAGGAAAACA and GAAAATTCCCCGTCGCTTCT. Control locus set 2: GGGTTCAGCCTCAACTTGTATCC and CCCACCACCTGGCATCTCT.

### The 90 kinase RT-PCR assay.

RNA was diluted to 300 μg/μl and treated with TURBO DNA-*free* (Ambion) following the manufacturer's protocol. cDNA was synthesized using random primers and SuperscriptII reverse transcription (Invitrogen) using the manufacturer's protocol. cDNA was amplified using gene-specific primers (concentration 5 μM) for the different human tyrosine kinases.

### Methylation detection.

Methylation of the DNA was detected using ApaI enzyme. DNA (2 μg) was dissolved in 50 μl of the appropriate buffer and 10 U of ApaI, and incubated at 30 °C overnight. A mirror condition was done in which the ApaI was exchanged by glycerol as a control for unspecific degradation. The resulting DNA was analyzed using two primer sets: one set spanning the restriction site (AATAAGACGTTGCAGAAGAG and TCGCAGAGCAAAAATTAAAGGGTGC) and another set next to the restriction site (CCCCGGGAGCATCTTGCAGCCA and TCGCAGAGCAAAAATTAAAGGGTGC).

Pull-down of methylated DNA fragments (restricted using EcoRI) was performed using the Methylcollector kit (Active Motif), and bisulfite modification of DNA was done using the EZ DNA Methylation-Gold kit (Zymo Research) according to the manufacturers' protocols.

### Array CGH.

We carried out array CGH using Code Linked Slides (AP Biotech) containing the 3,527 BAC clones from the Wellcome Trust Sanger Institute 1 Mb Clone Set, a gift from N. P. Carter (The Wellcome Trust Sanger Institute). Array CGH was performed as described [17].

### Sequencing.

The ATOH1 open reading frame was amplified with PCR using AATAAGACGTTGCAGAAGAG and TCGCAGAGCAAAAATTAAAGGGTGC and AmpliTaq Gold DNA polymerase and the GeneAmp PCR System 2400 (Applied Biosystems). The PCR products were purified and sequenced in both directions on the ABI Prism BigDye (Terminator Cycle Sequencing Kit version 1.1) on an ABI PRISM 3100 Genetic Analyser (Applied Biosystems).

## Supporting Information

Figure S1 Gut-Associated Lymphoid Tissues in AOM-Treated *Atoh1^wt^* Mice The colons of AOM-treated *Atoh1^wt^* mice showed large GALT that were macroscopically counted as polyps; shown is 5× magnification of representative colonic GALTs. GALT indicated by an arrow.
(726 KB PDF)

Figure S2 Histology of Polyps in *APC^min^* Background (A) Polyps in the *APC^min^* background still have goblet cells, indicating that *Atoh1* is still active.
(B) The polyps in the *APC^min^*; *Atoh1^Δintestin^e* mice originate in Atoh1 mutant tissue as seen by the absence of goblet cells.
(4.70 MB PDF)

Figure S3 Representative Normal-Appearing Crypts in AOM-Treated *Atoh1^wt^* and *Atoh1^Δintestine^* Colons AOM-treated colon sections with well-oriented crypts were used for BrdU and cleaved caspase-3 (c-Caspase 3) counting. The genotypes of the representative slides are indicated on the left side of the figure. The specific stain is identified at the top part of the figure. *Atoh1^wt^* (WT) crypts were distinguished from *Atoh1-null* crypts by the lack of the secretory goblet cells in the null crypts.
(A–C) Hematoxylin and eosin (H & E) staining of *Atoh1^wt^* crypts in *Atoh1^wt^* mice (A); and nondeleted *Atoh1^wt^* (B) and *Atoh1-null* (C) in *Atoh1Δintestine* mice.
(D–F) Representative BrdU staining of normal-appearing crypts in *Atoh1^wt^* mice (D) and nondeleted *Atoh1^wt^* (E) and *Atoh1-null* (F) in *Atoh1^Δintestine^* mice.
(G–I) Representative cleaved caspase-3 staining of normal-appearing crypts in *Atoh1^wt^* mice (G); and nondeleted *Atoh1^wt^* (H) and *Atoh1-null* (I) in *Atoh1^Δintestine^* mice. Arrows point to positive cells at the surface of the crypts. The images were captured at 20× magnification.
(4.96 MB PDF)

Figure S4 *ATOH1* Expression Correlates with Population Doubling Time (A) *ATOH1* mRNA transcripts in five independent MCC cell lines. The name of the cell line is indicated above each lane. RT-PCR was done with 100 ng of RNA under nonsaturating conditions.
(B) Doubling times in hours of the five MCC cell lines. Error bars indicate the standard deviation.
(C) RT-qPCR for *ATOH1* in MCC1 and MCC14.2 cell lines. *ATOH1* mRNA levels standardized to *GADPH* mRNA levels.
(760 KB PDF)

Figure S5 Summary Table of All Patient Data First column indicates patient numbers. Next, the relative ratios for *ATOH1* genomic DNA (gDNA) over control locus is presented for each of the two primer sets as analyzed by qPCR. The next two columns indicate the classification of the deletion/duplication status of the *ATOH1* locus. The mRNA expression is shown relative to control colon samples, with next to it, the classification of the expression. Clinical data are given in the third panel, namely cancer stage and metastasis. In the last panel, methylation of the *ATOH1* locus is shown using three different methods.
(760 KB PDF)

Figure S6 Array CGH Analysis of Three Patient Samples, Demonstrating No Aberration of the *ATOH1* Locus Each bar represents the log2 of the value for affected individuals (ind) versus a control sample (reference) for each probe, ordered based on the probes' chromosomal location. The region between 131 Mb and 141 Mb is shaded. The location of the *ATOH1* locus is shown with an arrow. The abnormalities were confirmed by dye swap experiments.
(4.55 MB PDF)

Figure S7 Methylation of the *ATOH1* Locus (A) Schematic representation of *ATOH1* locus. The white box indicate the *ATOH1* ORF, and the gray box the position of the CpG island. The primers are indicated as arrows.
(B) Detection of methylation at the *ATOH1* locus using the ApaI methylation-sensitive restriction enzyme. PCR fragments generated using primers spanning the ApaI restriction site from ApaI restricted genomic DNA; presence of a band indicates methylation of the *ATOH1* CpG island.
(C) Detection of methylation using methylation-sensitive PCR: upon bisulfite modification, presence of a band indicates methylation of the *ATOH1* CpG island.
(159 KB PDF)

Figure S8 Atoh1 Binds the Dnmt Proteins and Associates to a DNA Methyltransferase Activity (A) GST Pull-down assays using Atoh1 protein fused to GST (GST-Atoh1) and in vitro translated Dnmts (IVT-Dnmt1, IVT-Dnmt3a, or IVT-Dnmt3b).
(B) A GST-fused Atoh1 protein was used to purify DNA methyltransferase activity from nuclear extracts. After incubation, the beads were washed and assayed for DNA methyltransferase activity read as c.p.m. of S-adenosyl-l[methyl-3H] methionine incorporated into an oligonucleotide substrate. GST-tagged embryonic ectoderm development protein (GST-EED) was used as a positive control.
(C) Coimmunoprecipitation experiments shows that Atoh1 associated with Dnmt1. The 293T cells were transiently transfected in culture dishes (10-cm diameter) with 3 μg of HA-Atoh1 plasmid
(D) Mapping of Atoh1 binding to Dnmt1. GST Pull-downs were performed with Dnmt1 fragments fused to GST and in vitro–translated Atoh1. The upper part is a schematic representation of the Dnmt1 sequences used.
(1.04 MB PDF)

Figure S9 Immunohistochemical Analysis of Phosphorylated JNK in *Atoh1^Δintestine^* and *Atoh1^wt^* Mice (A) Representative pJNK1/2 staining in *Atoh1^wt^* crypt (dashed white line). The arrows indicate pJNK-positive cells.
(B) pJNK-positive cells (arrows) in wild-type (dashed gray line) and *Atoh1*-*null* (dashed black line) crypts in *Atoh1^Δintestine^* mice.
(C) Bar graph showing the percentage of pJNK-positive cells in wild-type mice (white); and wild-type (gray) and *Atoh1-null* crypts in *Atoh1^Δintestine^* mice. Error bars indicate the standard error of the mean. No significant differences between genotypes were detected.
(4.18 MB PDF)

Figure S10 ATOH1 Interacts with the Cell Cycle and Apoptosis (A) Cell cycle distribution of MCC14.2-derived cell lines without (MCC14.2 and MCC14.2-GFP) and with *Atoh1* expression (MCC14.2-Atoh1.1a and MCC14.2-Atoh1.2a). No significant change in distribution throughout the cell cycle can be observed.
(B) Maximal projection image of AnnexinV staining (red) on cells transduced with lentiviral vectors expressing GFP (left panel) or Atoh1-IRES-GFP (right panel). GFP is in green.
(C) Western blot analysis for CyclinA1, PCNA, p27^kip^, c-myc, phospho-H3 and p21^waf1^ of lysates of MCC14.2 cells, MCC14.2-GFP and two MCC14.2 cell lines transduced with *Atoh1*-IRES-*eGFP* (MCC14.2-Atoh1.1a and MCC14.2-Atoh1.2a). The corresponding actin loading controls are shown under each blot.
(D–H) Quantification of expression levels of cyclinA1 (D), PCNA (E), p27 (F), c-myc (G), and phospho-histoneH3. (H) Representative blots are shown in (C).
(2.90 MB PDF)

Figure S11 ATOH1 Acts Independently of Notch but Modulates RTK Expression (A) RT-PCR for target of Notch signaling HES1 on mRNA isolated from MCC14.2 cells, MCC14.2 cells transduced with GFP, and two MCC14.2 cell lines transduced with *Atoh1*-IRES-*eGFP* (MCC14.2-Atoh1.1a and MCC14.2-Atoh1.2a). GADPH loading control is shown below.
(B) Western blot analysis for cleaved intracellular Notch (NICD). Different concentrations of presinilin inhibitor X were used; a plus sign (+) indicates a positive control for cleaved NICD.
(C) Different concentrations of γ-secretase inhibitor (inhibitor X) on MCC14.2 do not influence the proliferation rate. First lane: 10 μM inhibitor X, second lane: DMSO control of previous lane, third lane: 1 μM inhibitor X, fourth lane: DMSO control of previous lane, and fifth lane: untreated.
(D) RT-PCR for expression of 90 tyrosine kinases scored from undetectable (white) over orange (expression) to red (strong expression) on mRNA or on untransduced MCC14.2 cells (lane 1), GFP transduced MCC14.2 cell line (lane 2), and two independent MCC14.2-derived cell lines transduced with *Atoh1*-IRES-*eGFP* vectors (lane 3: MCC14.2-Atoh1.2a) and on untransfected HT29 cells (lane14: HT29), GFP-transfected HT29 cells (lane5: HT29-GFP), and HT29 cells transfected with the *Atoh1*-IRES-*eGFP* construct (lane 6: HT29-Atoh1).
(E) RT-qPCR for *ATOH1* mRNA levels (compared to GADPH mRNA levels) in MCC cell lines. MCC1 (first lane: MCC cell line with endogenous high *ATOH1* expression), MCC14.2 (lane 2), GFP-transduced MCC14.2 cell line (lane 3), and two independently made MCC14.2-derived cell lines transduced with Atoh1-IRES-GFP vectors (lane 4: MCC14.2-Atoh1.1a, and lane 5: MCC14.2-Atoh1.2a).
(F) Western blot analysis for NTRK1 and Neurotrophin-3 (NT3) of untransduced MCC14.2 cells (lane 1), GFP-transduced MCC14.2 cell line (lane 2), and two independently made MCC14.2-derived cell lines transduced with Atoh1-IRES-GFP vectors (lane 3: MCC14.2-Atoh1.1a, and lane 4: MCC14.2-Atoh1.2a); actin loading controls are represented below each blot.
(G and H) Quantifications of western blots in (F), normalized to actin.
(2.04 MB PDF)

Figure S12 Quantifications of Western Blot Analysis in Main Figures (Minimum of Two Blots per Quantification) The quantifications are performed with USI software. The signal for the protein of interest was standardized to its respective actin loading control. Antibody is stated above each graph, representative experiments are shown in main figures: (A) Figure 4B, (B) Figure 4B, (C) Figure 4B, (D1) Figure 4C, (D1′) Figure 4C, (D2) Figure 4C, (D2′) Figure 4C, (E) Figure 5B , (F) Figure 5B, (G) Figure 6A, (H) Figure 6A, (I) Figure 6A, (J) Figure 6A, (K) Figure 6B, (L) Figure 6B, (M) Figure 6B, (N) Figure 6B, (O) Figure 6C, (P) Figure 6C, (Q) Figure 6C, (R) Figure 6E, (S) Figure 6E, (T) Figure 6E, (U) Figure 7D, (V) Figure 7D, (W) Figure 7E, (X) Figure 7E, and (Y) Figure 7E.
(365 KB PDF)

## Abbreviations

AOM: azoxymethane
CGH: comparative genomic hybridization
CRC: colorectal cancer
GALT: gut-associated lymphoid tissue
JNK: Jun N-terminal kinase
MCC: Merkel cell carcinoma
NTRK: Neurotrophic tyrosine kinase receptor type 1
RTK: receptor tyrosine kinase
RT-qPCR: quantitative reverse-transcriptase PCR
