# Supplementary material for: Atonal homolog 1 Is a Tumor Suppressor Gene
Source: PLoS Biol. 2009 Feb 24;7(2):e1000039. doi: 10.1371/journal.pbio.1000039 (PMC2652388; doi:10.1371/journal.pbio.1000039)
Supplement: Figure S1 — The colons of AOM-treated Atoh1wt mice showed large GALT that were macroscopically counted as polyps; shown is 5× magnification of representative colonic GALTs. GALT indicated by an arrow. (726 KB PDF) [file pbio.1000039.sg001.pdf]

Atonal homologue 1 is a tumour suppressor gene

Bossuyt *et al.*

Supplementary figures

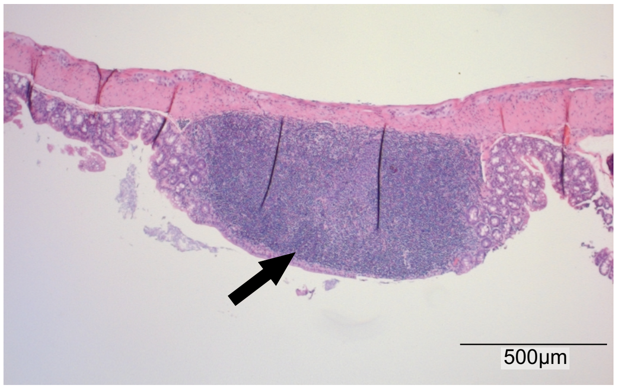

Supplementary figure 1: Gut associated lymphoid tissues in AOM-treated *Atoh1*^{wt} mice. The colons of AOM-treated *Atoh1*^{wt} mice showed large gut associated lymphoid tissues (GALT) that were macroscopically counted as polyps. 5x magnification of representative colonic GALTs. Galt indicated by an arrow.
