# Supplementary material for: Atonal homolog 1 Is a Tumor Suppressor Gene
Source: PLoS Biol. 2009 Feb 24;7(2):e1000039. doi: 10.1371/journal.pbio.1000039 (PMC2652388; doi:10.1371/journal.pbio.1000039)
Supplement: Figure S2 — (A) Polyps in the APCmin background still have goblet cells, indicating that Atoh1 is still active. (B) The polyps in the APCmin; Atoh1Δintestine mice originate in Atoh1 mutant tissue as seen by the absence of goblet cells. (4.70 MB PDF) [file pbio.1000039.sg002.pdf]

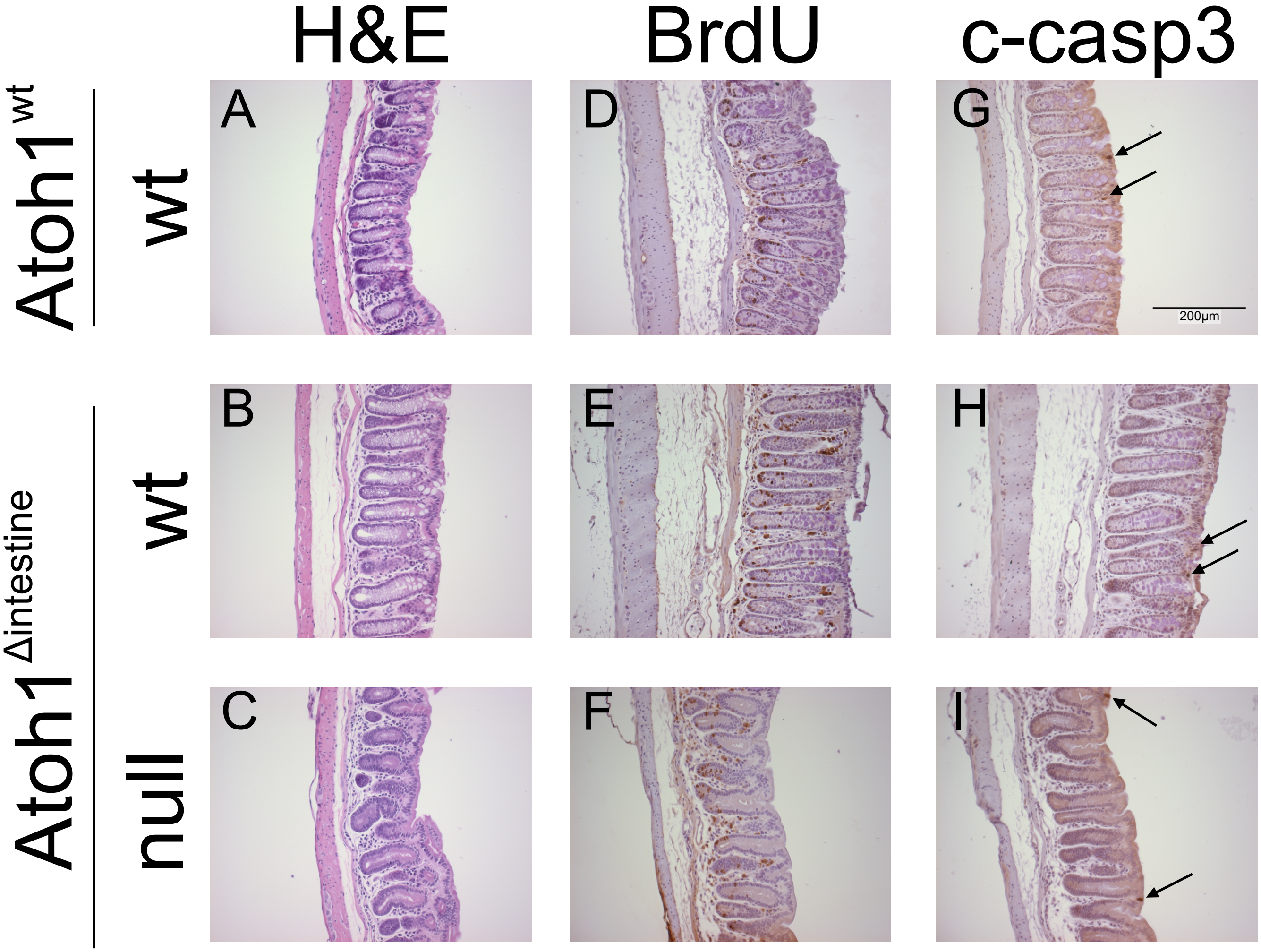

Supplementary figure 2: Representative normal-appearing crypts in AOM-treated *Atoh1*^{wt} **and** *Atoh1*$^{\Delta intestine}$ **colons**. AOM-treated colon sections with well oriented crypts were used for BrdU and cleaved caspase-3 (c-Caspase 3) counting. The genotypes of the representative slides are indicated on the left side of the figure. The specific stain is identified at the top part of the figure. *Atoh1*^{wt} (WT) crypts were distinguished from *Atoh1-null* crypts by the lack of the secretory goblet cells in the null crypts. **A-C**. Hematoxylin and eosin (H & E) staining of *Atoh1*^{wt} crypts in *Atoh1*^{wt} mice (**A**); and non-deleted *Atoh1*^{wt} (**B**) and *Atoh1-null* (**C**) in *Atoh1*$^{\Delta intestine}$ mice. **D-F**. Representative BrdU staining of normal-appearing crypts in *Atoh1*^{wt} mice (**D)** and non-deleted *Atoh1*^{wt} (**E**) and *Atoh1-null* (**F**) in *Atoh1*$^{\Delta intestine}$ mice. **G-I**. Representative cleaved caspase-3 staining of normal-appearing crypts in *Atoh1*^{wt} mice (**G**); and non-deleted *Atoh1*^{wt} (**H**) and *Atoh1-null* (**I**) in *Atoh1*$^{\Delta intestine}$ mice. Arrows point to positive cells at the surface of the crypts. The images were captured at 20x magnification.
