# Supplementary material for: Atonal homolog 1 Is a Tumor Suppressor Gene
Source: PLoS Biol. 2009 Feb 24;7(2):e1000039. doi: 10.1371/journal.pbio.1000039 (PMC2652388; doi:10.1371/journal.pbio.1000039)
Supplement: Figure S3 — AOM-treated colon sections with well-oriented crypts were used for BrdU and cleaved caspase-3 (c-Caspase 3) counting. The genotypes of the representative slides are indicated on the left side of the figure. The specific stain is identified at the top part of the figure. Atoh1wt (WT) crypts were distinguished from Atoh1-null crypts by the lack of the secretory goblet cells in the null crypts. (A–C) Hematoxylin and eosin (H & E) staining of Atoh1wt crypts in Atoh1wt mice (A); and nondeleted Atoh1wt (B) and Atoh1-null (C) in Atoh1Δintestine mice. (D–F) Representative BrdU staining of normal-appearing crypts in Atoh1wt mice (D) and nondeleted Atoh1wt (E) and Atoh1-null (F) in Atoh1Δintestine mice. (G–I) Representative cleaved caspase-3 staining of normal-appearing crypts in Atoh1wt mice (G); and nondeleted Atoh1wt (H) and Atoh1-null (I) in Atoh1Δintestine mice. Arrows point to positive cells at the surface of the crypts. The images were captured at 20× magnification. (4.96 MB PDF) [file pbio.1000039.sg003.pdf]

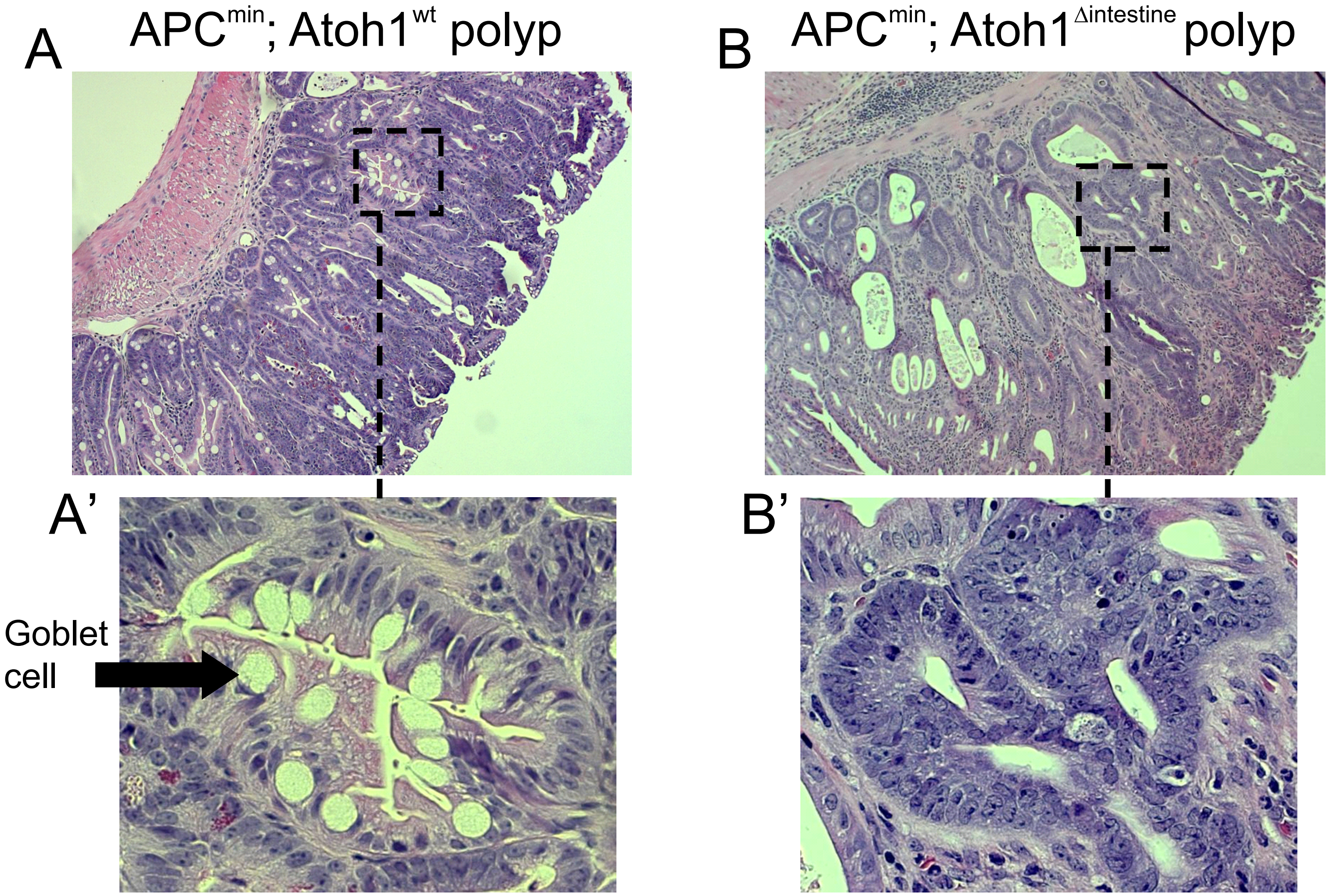

Supplementary figure 2: Histology of polyps in APC^{min} background. A, Polyps in the APCmin background still have goblet cells, indicating that Atoh1 is still active. **B,** The polyps in the APC^{min}; $Atoh1^{\Delta intestine}$ mice originate in Atoh1 mutant tissue as seen by the absence of goblet cells.
