# Supplementary material for: Atonal homolog 1 Is a Tumor Suppressor Gene
Source: PLoS Biol. 2009 Feb 24;7(2):e1000039. doi: 10.1371/journal.pbio.1000039 (PMC2652388; doi:10.1371/journal.pbio.1000039)
Supplement: Figure S4 — (A) ATOH1 mRNA transcripts in five independent MCC cell lines. The name of the cell line is indicated above each lane. RT-PCR was done with 100 ng of RNA under nonsaturating conditions. (B) Doubling times in hours of the five MCC cell lines. Error bars indicate the standard deviation. (C) RT-qPCR for ATOH1 in MCC1 and MCC14.2 cell lines. ATOH1 mRNA levels standardized to GADPH mRNA levels. (760 KB PDF) [file pbio.1000039.sg004.pdf]

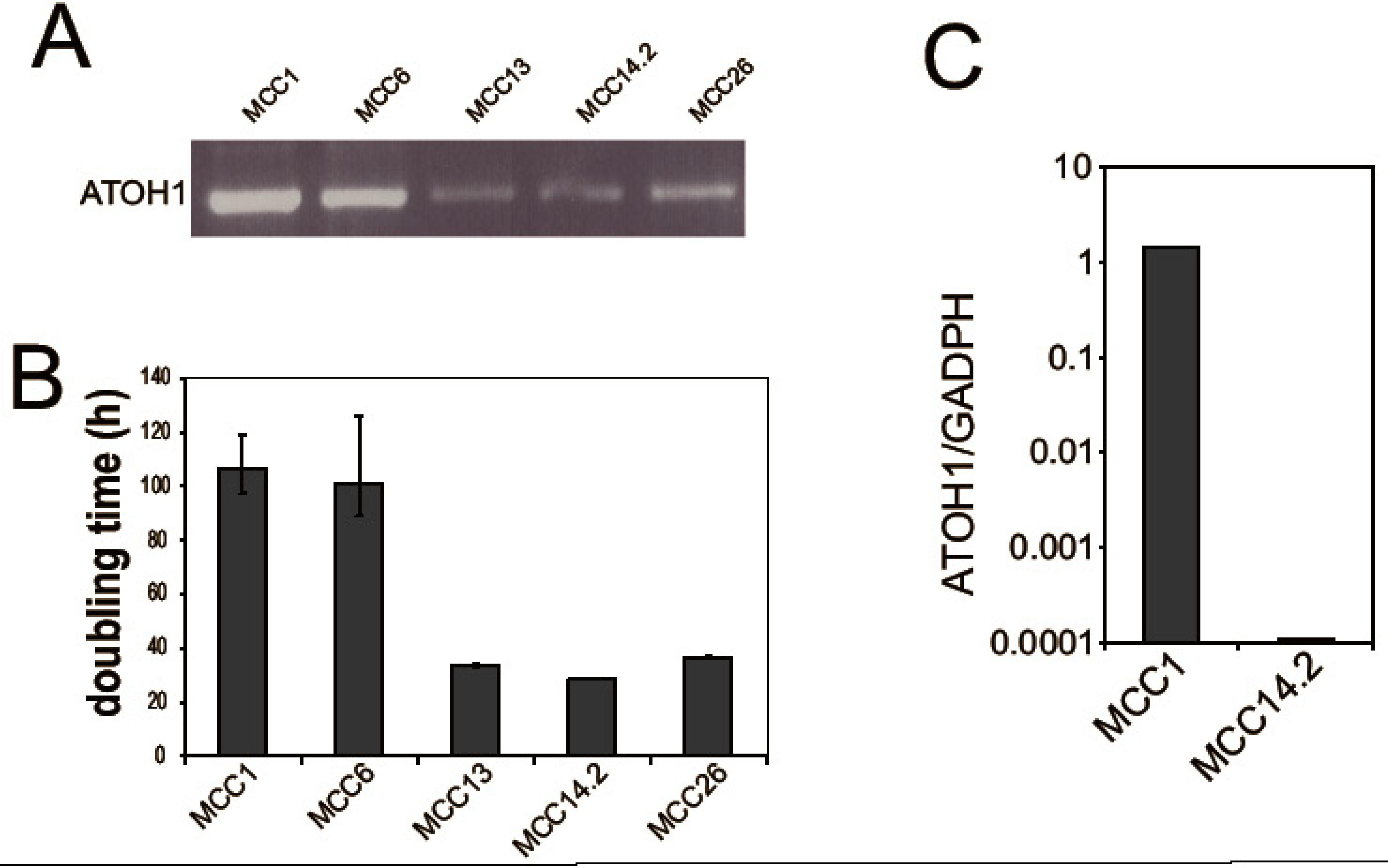

Supplementary Figure 4: A, *ATOH1* mRNA transcripts in 5 independent MCC cell lines. The name of the cell line is indicated above each lane. RT-PCR was done with 100 ng of RNA under non-saturating conditions. **B,** Doubling times in hours of the 5 MCC cell lines. Error bars are standard deviation. **C,** RT-qPCR for *ATOH1* in MCC1 and MCC14.2 cell lines. *ATOH1* mRNA levels standardised to *GADPH* mRNA levels.
