# Supplementary material for: Atonal homolog 1 Is a Tumor Suppressor Gene
Source: PLoS Biol. 2009 Feb 24;7(2):e1000039. doi: 10.1371/journal.pbio.1000039 (PMC2652388; doi:10.1371/journal.pbio.1000039)
Supplement: Figure S5 — First column indicates patient numbers. Next, the relative ratios for ATOH1 genomic DNA (gDNA) over control locus is presented for each of the two primer sets as analyzed by qPCR. The next two columns indicate the classification of the deletion/duplication status of the ATOH1 locus. The mRNA expression is shown relative to control colon samples, with next to it, the classification of the expression. Clinical data are given in the third panel, namely cancer stage and metastasis. In the last panel, methylation of the ATOH1 locus is shown using three different methods. (760 KB PDF) [file pbio.1000039.sg005.pdf]

patient	primer set 1	primer set 2	primer set 1	primer set 2	mRNA expression		microscopy	metastasis	methylcollector	ApaI	bisulfite PCR
1	0.059	0.826	2 deletions	no deletion	0.066	low	adenocarcinoma	no	methylated	methylated	no
2	0.485	0.655	1 deletion	1 deletion	0.082	low	adenocarcinoma	no	methylated	methylated	methylated
3	0.285	0.841	1 deletion	no deletion	0.092	low	adenocarcinoma	no	methylated	methylated	methylated
4	0.076	0.590	2 deletions	1 deletion	0.280	normal	adenocarcinoma	no	methylated	no	no
5	1.434	1.474	1 duplication	1 duplication	0.018	low	adenocarcinoma	no	methylated	methylated	methylated
6	0.390	1.248	1 deletion	no deletion	0.265	normal	adenoma	no	methylated	methylated	methylated
7	0.983	0.847	no deletion	no deletion	0.167	low	adenocarcinoma	no	methylated	methylated	no
8	1.464	1.061	1 duplication	no deletion	0.191	low	adenocarcinoma	no	methylated	no	no
9	1.449	1.310	1 duplication	1 duplication	0.267	normal	adenocarcinoma	no	no	no	no
10	0.143	0.388	2 deletions	1 deletion	0.286	normal	adenocarcinoma	no	no	no	no
11	1.905	1.275	2 duplications	no deletion	0.104	low	adenocarcinoma	no	methylated	methylated	methylated
12	0.512	1.301	1 deletion	1 duplication	0.016	low	adenocarcinoma	yes	methylated	methylated	methylated
13	0.030	0.457	2 deletions	1 deletion	0.146	low	adenocarcinoma	no	methylated	no	no
14	2.196	1.366	2 duplications	1 duplication	0.003	low	adenocarcinoma	yes	no	no	no
15	1.324	0.908	1 duplication	no deletion	0.039	low	adenocarcinoma	no	methylated	methylated	no
16	1.459	0.498	1 duplication	1 deletion	0.354	normal	adenocarcinoma	no	no	no	no
17	0.338	0.265	1 deletion	1 deletion	0.009	low	adenoma	no	methylated	no	no
18	0.969	0.662	no deletion	1 deletion	3.138	high	adenoma	no	methylated	methylated	methylated
19	0.383	0.500	1 deletion	1 deletion	0.045	low	adenocarcinoma	no	methylated	methylated	methylated
20	0.516	0.737	1 deletion	1 deletion	0.013	low	adenocarcinoma	yes	no	no	no
21	0.351	0.505	1 deletion	1 deletion	0.387	normal	adenoma	no	methylated	methylated	no
22	0.782	0.538	no deletion	1 deletion	0.218	low	adenocarcinoma	no	methylated	no	no
23	0.363	0.476	1 deletion	1 deletion	1.803	high	adenocarcinoma	no	methylated	methylated	no
24	0.281	0.332	1 deletion	1 deletion	0.966	normal	adenoma	no	methylated	methylated	no
25	0.480	0.821	1 deletion	no deletion	0.470	normal	adenoma	no	methylated	no	no
26	0.904	0.473	no deletion	1 deletion	0.008	low	adenocarcinoma	yes	methylated	methylated	methylated
27	1.625	0.500	1 duplication	1 deletion	0.455	normal	adenoma	yes	methylated	methylated	methylated
28	0.455	0.437	1 deletion	1 deletion	0.354	normal	adenoma	yes	methylated	no	no
29	2.000	3.719	2 duplications	mult duplications	0.134	low	adenocarcinoma	yes	methylated	methylated	no
30	2.630	3.215	mult duplications	mult duplications	73.772	high	adenocarcinoma	yes	methylated	methylated	methylated
31	1.227	0.588	no deletion	1 deletion	0.000	low	adenocarcinoma	yes	no	no	no
32	0.026	0.158	2 deletions	2 deletions	0.006	low	adenocarcinoma	no	methylated	no	methylated
33	1.185	0.745	no deletion	1 deletion	0.000	low	adenocarcinoma	no	methylated	no	methylated
34	1.608	0.529	1 duplication	1 deletion	0.007	low	adenocarcinoma	no	no	methylated	no
35	2.387	1.925	mult duplications	2 duplications	0.032	low	adenocarcinoma	no	methylated	no	no
36	2.990	2.732	mult duplications	mult duplications	0.000	low	adenocarcinoma	no	methylated	no	no
37	1.796	1.586	2 duplications	1 duplication	0.003	low	adenocarcinoma	yes	no	methylated	no
38	0.771	0.997	no deletion	no deletion	0.068	low	adenocarcinoma	yes	methylated	methylated	no
39	0.674	1.840	1 deletion	2 duplications	0.047	low	adenoma	yes	no	no	no
40	0.745	0.712	no deletion	1 deletion	0.022	low	adenoma	yes	methylated	no	methylated
41	0.601	0.351	1 deletion	1 deletion	0.046	low	adenocarcinoma	yes	methylated	methylated	no
42	0.168	0.307	2 deletions	1 deletion	0.000	low	adenocarcinoma	yes	methylated	methylated	methylated
Ht29	0.871	0.435	no deletion	1 deletion	0.007	low	adenocarcinoma	yes	methylated	no	no
44	1.149	0.633	no deletion	1 deletion	**0.0378		MCC	yes	methylated	methylated	methylated
45	0.529	0.798	1 deletion	no deletion	**1.591		MCC	no	methylated	methylated	no
46	0.500	0.343	1 deletion	1 deletion	**4.5		MCC	no	methylated	methylated	methylated
MCC14.2	0.365	0.471	1 deletion	1 deletion	**0.004		MCC	yes	methylated	methylated	no
control	1.000	1.000	no deletion	no deletion	1 ± 0.374	normal	normal gut	no	no		no

Supplemental figure 5: summary table of all patient data. First column indicates patient numbers. Next the relative ratios for ATOH1 gDNA over control locus is presented for each of the two primer sets as analysed by qPCR. The next two collumns indicate the classification of the deletion/duplication status of the ATOH1 locus.
The mRNA expression is shown relative to control colon samples, with next to it the classification of the expression.
Clinical data are give in the third panel, namely cancer stage and metastasis.
In the last panel, methylation of the ATOH1 locus is shown using three different methods.
