# Supplementary material for: Atonal homolog 1 Is a Tumor Suppressor Gene
Source: PLoS Biol. 2009 Feb 24;7(2):e1000039. doi: 10.1371/journal.pbio.1000039 (PMC2652388; doi:10.1371/journal.pbio.1000039)
Supplement: Figure S6 — Each bar represents the log2 of the value for affected individuals (ind) versus a control sample (reference) for each probe, ordered based on the probes' chromosomal location. The region between 131 Mb and 141 Mb is shaded. The location of the ATOH1 locus is shown with an arrow. The abnormalities were confirmed by dye swap experiments. (4.55 MB PDF) [file pbio.1000039.sg006.pdf]

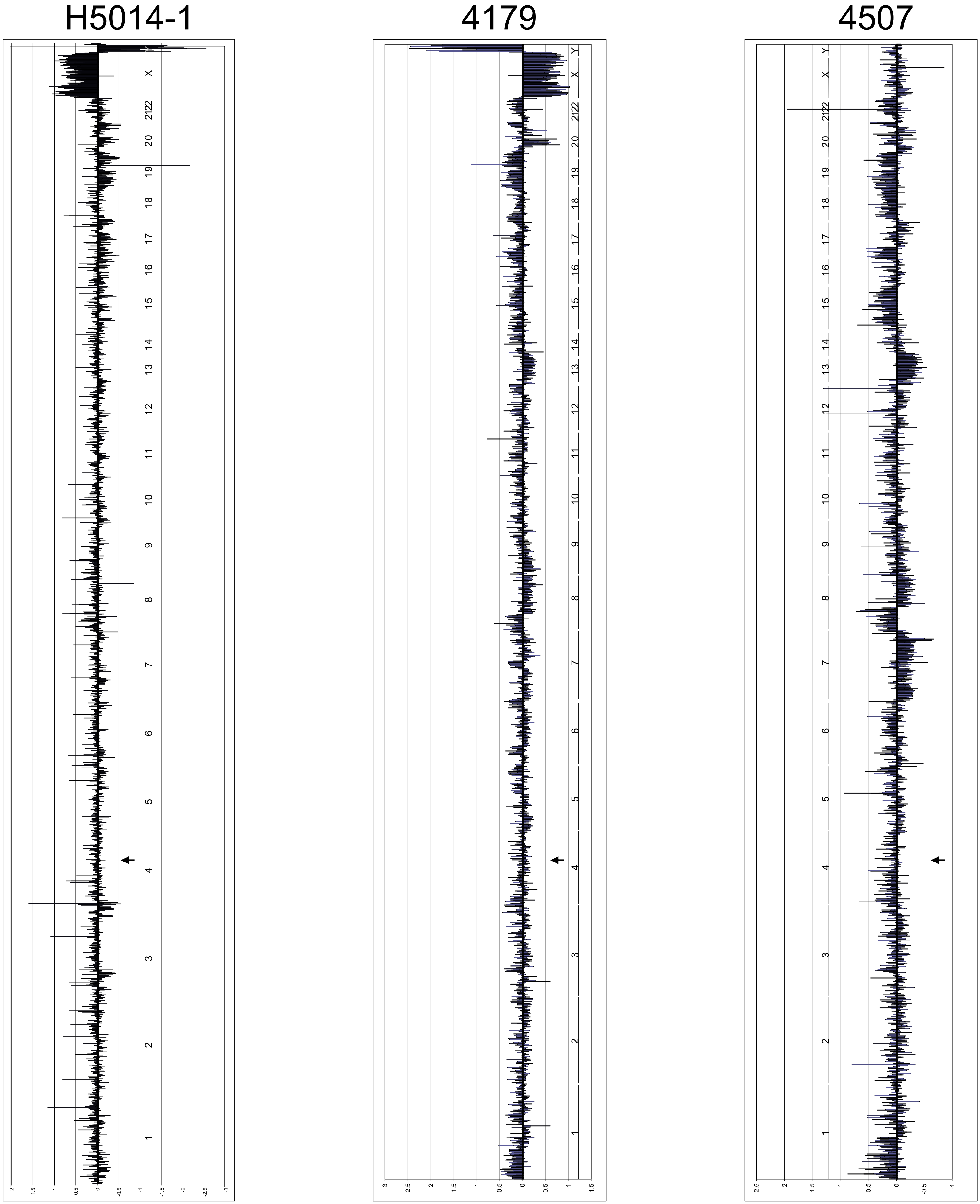


**Supplementary Figure 6:** Array CGH analysis of three patient samples, demonstrating no aberration of the *ATOH1* locus. Each bar represents the log2 of the value for affected individuals ('ind') versus a control sample ('reference') for each probe, ordered based on the probes' chromosomal location. The region between 131 Mb and 141 Mb is shaded. The location of the *ATOH1* locus is shown with an arrow. The abnormalities were confirmed by dye swap experiments.
