# Supplementary material for: Atonal homolog 1 Is a Tumor Suppressor Gene
Source: PLoS Biol. 2009 Feb 24;7(2):e1000039. doi: 10.1371/journal.pbio.1000039 (PMC2652388; doi:10.1371/journal.pbio.1000039)
Supplement: Figure S7 — (A) Schematic representation of ATOH1 locus. The white box indicate the ATOH1 ORF, and the gray box the position of the CpG island. The primers are indicated as arrows. (B) Detection of methylation at the ATOH1 locus using the ApaI methylation-sensitive restriction enzyme. PCR fragments generated using primers spanning the ApaI restriction site from ApaI restricted genomic DNA; presence of a band indicates methylation of the ATOH1 CpG island. (C) Detection of methylation using methylation-sensitive PCR: upon bisulfite modification, presence of a band indicates methylation of the ATOH1 CpG island. (159 KB PDF) [file pbio.1000039.sg007.pdf]

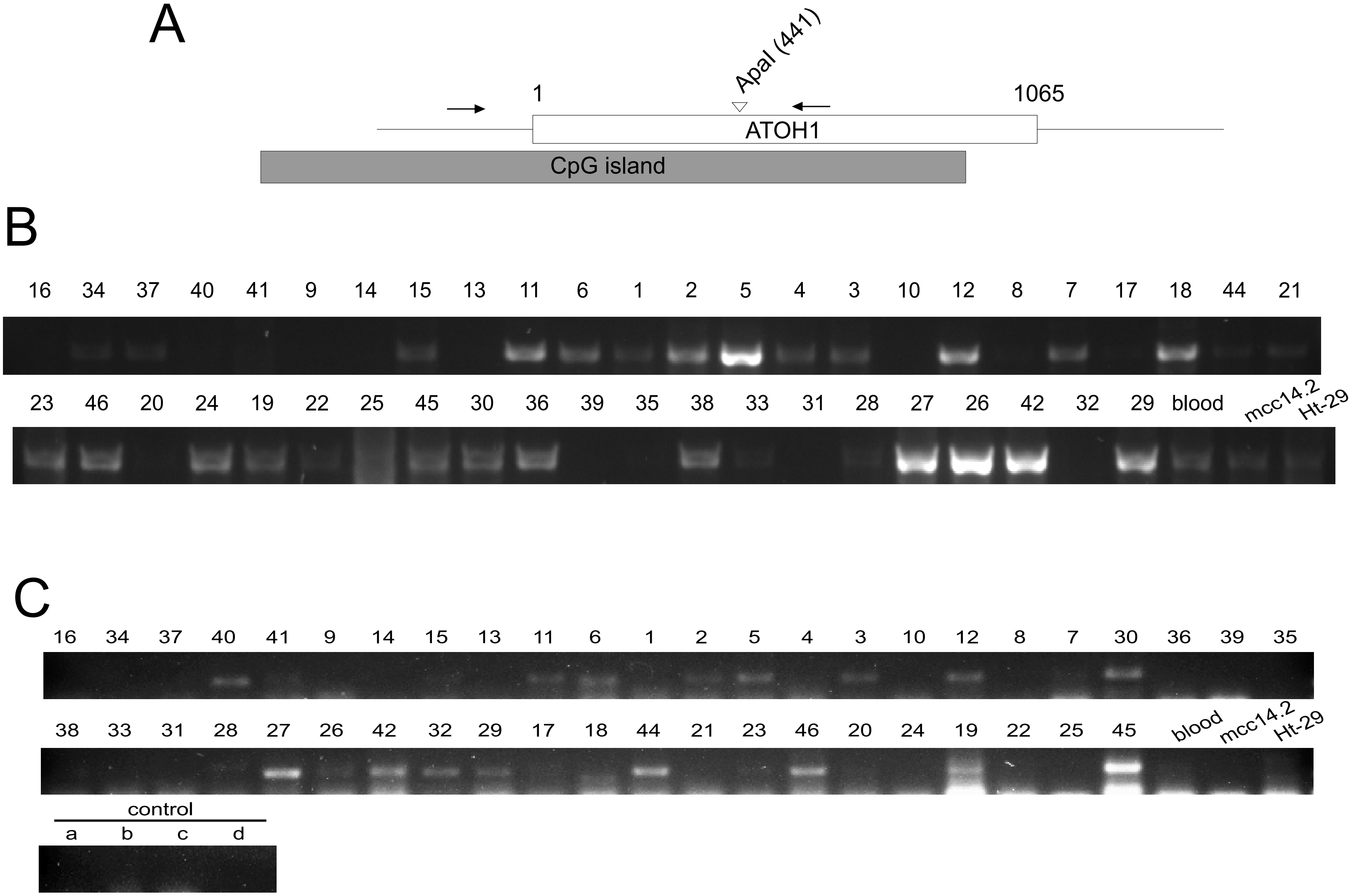

Supplementary Figure 7 : A, Schematic representation of *ATOH1* locus. White box indicate the *ATOH1* ORF and gray box the position of the CpG island. The primers are indicated as arrows. **B,** Detection of methylation at the *ATOH1* locus using the ApaI methylation sensitive restriction enzyme. PCR fragments generated using primers spanning the ApaI restriction site from ApaI restricted genomic DNA, presence of a band indicates methylation of the Atoh1 CpG island. **C,** Detection of methylation using methylation sensitive PCR, upon bisulfite modification, presence of a band indicates methylation of the Atoh1 CpG island..
