# Supplementary material for: Atonal homolog 1 Is a Tumor Suppressor Gene
Source: PLoS Biol. 2009 Feb 24;7(2):e1000039. doi: 10.1371/journal.pbio.1000039 (PMC2652388; doi:10.1371/journal.pbio.1000039)
Supplement: Figure S8 — (A) GST Pull-down assays using Atoh1 protein fused to GST (GST-Atoh1) and in vitro translated Dnmts (IVT-Dnmt1, IVT-Dnmt3a, or IVT-Dnmt3b). (B) A GST-fused Atoh1 protein was used to purify DNA methyltransferase activity from nuclear extracts. After incubation, the beads were washed and assayed for DNA methyltransferase activity read as c.p.m. of S-adenosyl-l[methyl-3H] methionine incorporated into an oligonucleotide substrate. GST-tagged embryonic ectoderm development protein (GST-EED) was used as a positive control. (C) Coimmunoprecipitation experiments shows that Atoh1 associated with Dnmt1. The 293T cells were transiently transfected in culture dishes (10-cm diameter) with 3 μg of HA-Atoh1 plasmid (D) Mapping of Atoh1 binding to Dnmt1. GST Pull-downs were performed with Dnmt1 fragments fused to GST and in vitro–translated Atoh1. The upper part is a schematic representation of the Dnmt1 sequences used. (1.04 MB PDF) [file pbio.1000039.sg008.pdf]

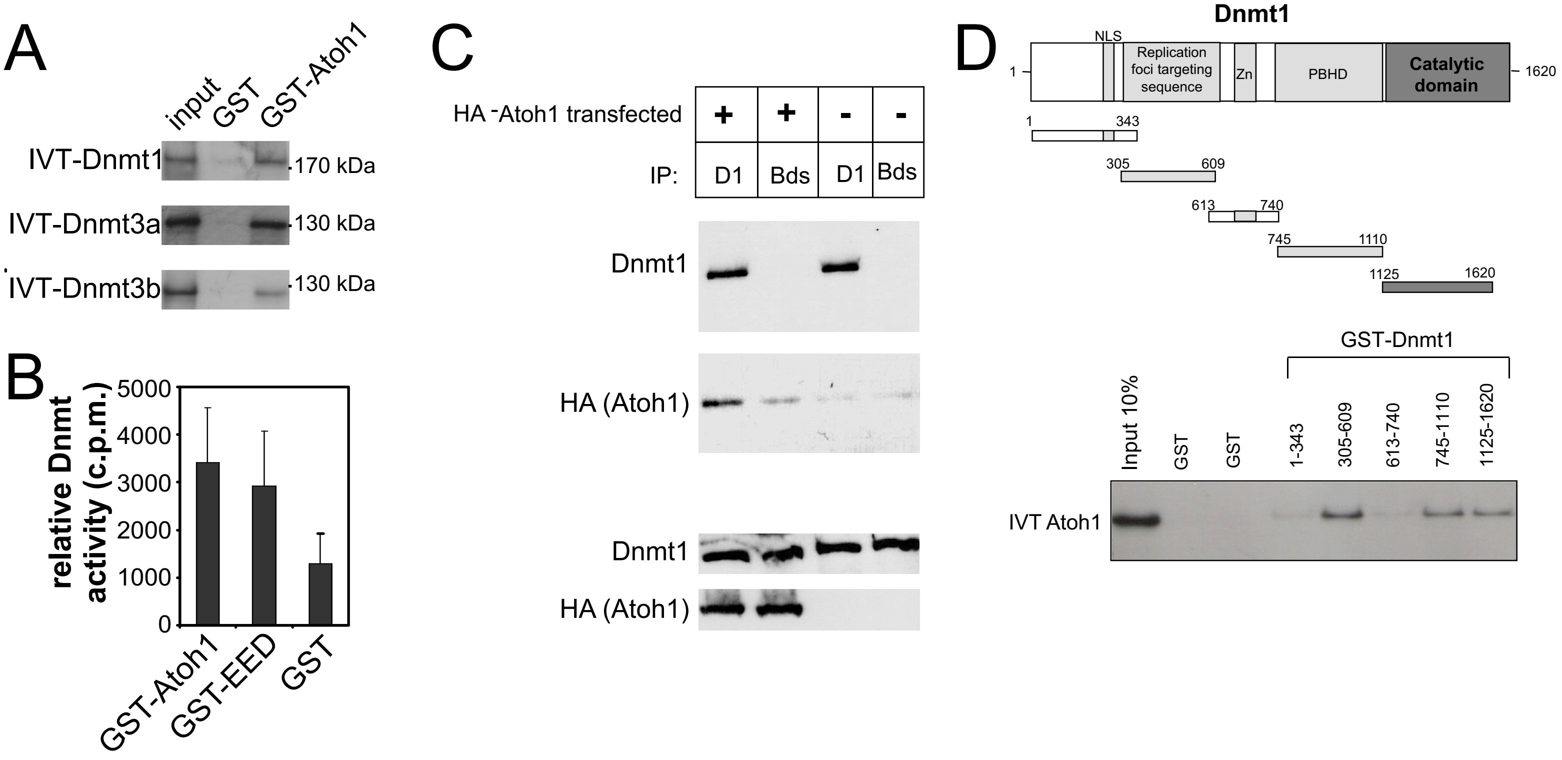

Supplementary Figure 8: Atoh1 binds the DNMT proteins and associates to a DNA methyltransferase activity. **A,** GST Pull-down assays using Atoh1 protein fused to GST (GST-Atoh1) and *in vitro* translated Dnmts (IVT-Dnmt1, IVT-Dnmt3a or IVT-Dnmt3b). **B,** A GST-fused Atoh1 protein was used to purify DNA methyltransferase activity from nuclear extracts. After incubation, the beads were washed and assayed for DNA methyltransferase activity read as c.p.m. of S-adenosyl-L[methyl-3H] methionine incorporated into an oligonucleotide substrate. GST-tagged embryonic ectoderm development protein (GST-EED) was used as a positive control. **C,** Co-Immunoprecipitation experiments shows that Atoh1 associated with Dnmt1. 293T cells were transiently transfected in culture dishes (10 cm diameter) with 3 µg of HA-Atoh1 plasmid **D,** Mapping of Atoh1 binding to Dnmt1. GST Pull-downs were performed with Dnmt1 fragments fused to GST and in vitro translated Atoh1. The upper part is a schematic representation of the Dnmt1 sequences used.
