# Supplementary material for: Atonal homolog 1 Is a Tumor Suppressor Gene
Source: PLoS Biol. 2009 Feb 24;7(2):e1000039. doi: 10.1371/journal.pbio.1000039 (PMC2652388; doi:10.1371/journal.pbio.1000039)
Supplement: Figure S9 — (A) Representative pJNK1/2 staining in Atoh1wt crypt (dashed white line). The arrows indicate pJNK-positive cells. (B) pJNK-positive cells (arrows) in wild-type (dashed gray line) and Atoh1-null (dashed black line) crypts in Atoh1Δintestine mice. (C) Bar graph showing the percentage of pJNK-positive cells in wild-type mice (white); and wild-type (gray) and Atoh1-null crypts in Atoh1Δintestine mice. Error bars indicate the standard error of the mean. No significant differences between genotypes were detected. (4.18 MB PDF) [file pbio.1000039.sg009.pdf]

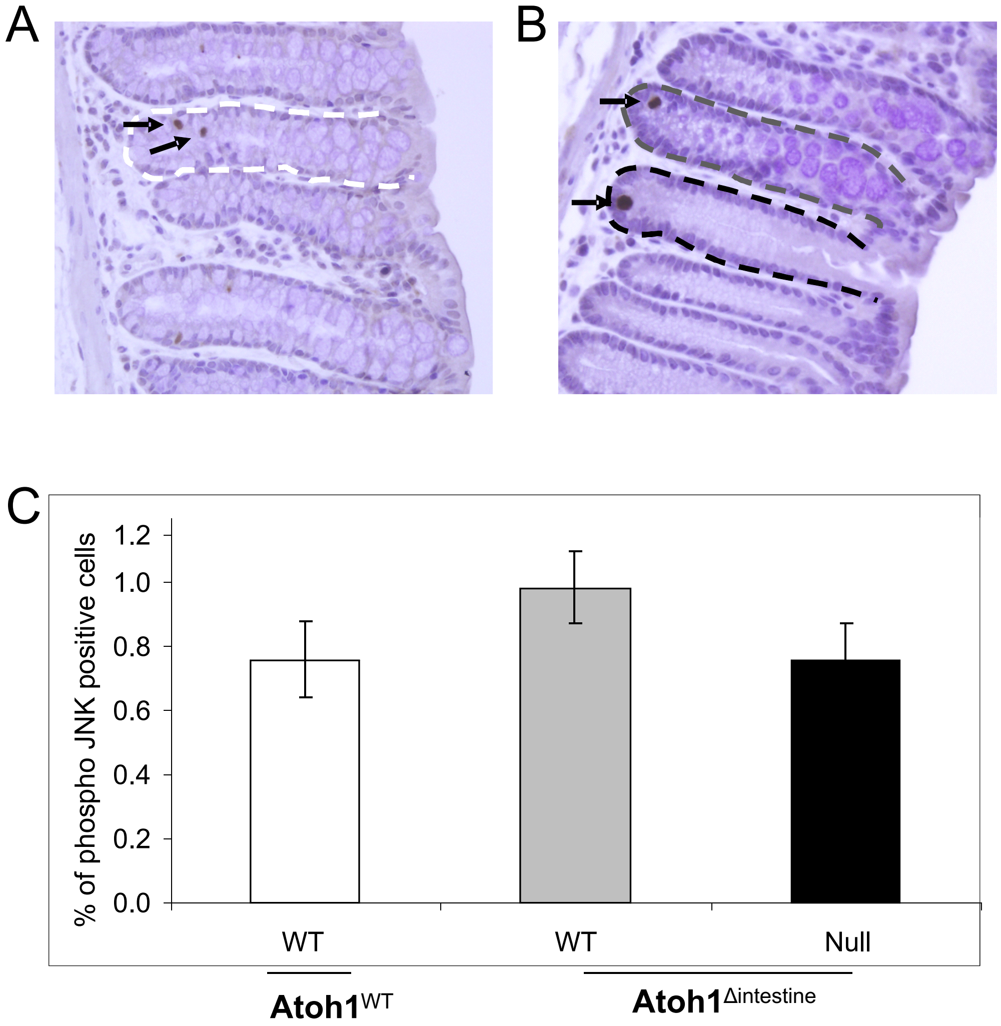

Supplemental Figure 9: Immunohistochemical analysis of phosphorylated JNK in Atoh1$^{\square intestine}$ and Atoh1^{WT} mice. **A,** Representative pJNK1/2 staining in Atoh1^{WT} crypt (dashed white line). The arrows indicate pJNK positive cells. **B,** pJNK positive cells (arrows) in wild type (dashed gray line) and Atoh1 null (dashed black line) crypts in Atoh1$^{\square intestine}$ mice. **C,** Bar graph showing the percentage of pJNK positive cells in wild type mice (white); wild type (gray) and Atoh1 null crypts in Atoh1$^{\square intestine}$ mice. Error bars show standard error of the mean. No significant differences between genotypes was detected.
