# Supplementary material for: Atonal homolog 1 Is a Tumor Suppressor Gene
Source: PLoS Biol. 2009 Feb 24;7(2):e1000039. doi: 10.1371/journal.pbio.1000039 (PMC2652388; doi:10.1371/journal.pbio.1000039)
Supplement: Figure S10 — (A) Cell cycle distribution of MCC14.2-derived cell lines without (MCC14.2 and MCC14.2-GFP) and with Atoh1 expression (MCC14.2-Atoh1.1a and MCC14.2-Atoh1.2a). No significant change in distribution throughout the cell cycle can be observed. (B) Maximal projection image of AnnexinV staining (red) on cells transduced with lentiviral vectors expressing GFP (left panel) or Atoh1-IRES-GFP (right panel). GFP is in green. (C) Western blot analysis for CyclinA1, PCNA, p27kip, c-myc, phospho-H3 and p21waf1 of lysates of MCC14.2 cells, MCC14.2-GFP and two MCC14.2 cell lines transduced with Atoh1-IRES-eGFP (MCC14.2-Atoh1.1a and MCC14.2-Atoh1.2a). The corresponding actin loading controls are shown under each blot. (D–H) Quantification of expression levels of cyclinA1 (D), PCNA (E), p27 (F), c-myc (G), and phospho-histoneH3. (H) Representative blots are shown in (C). (2.90 MB PDF) [file pbio.1000039.sg010.pdf]

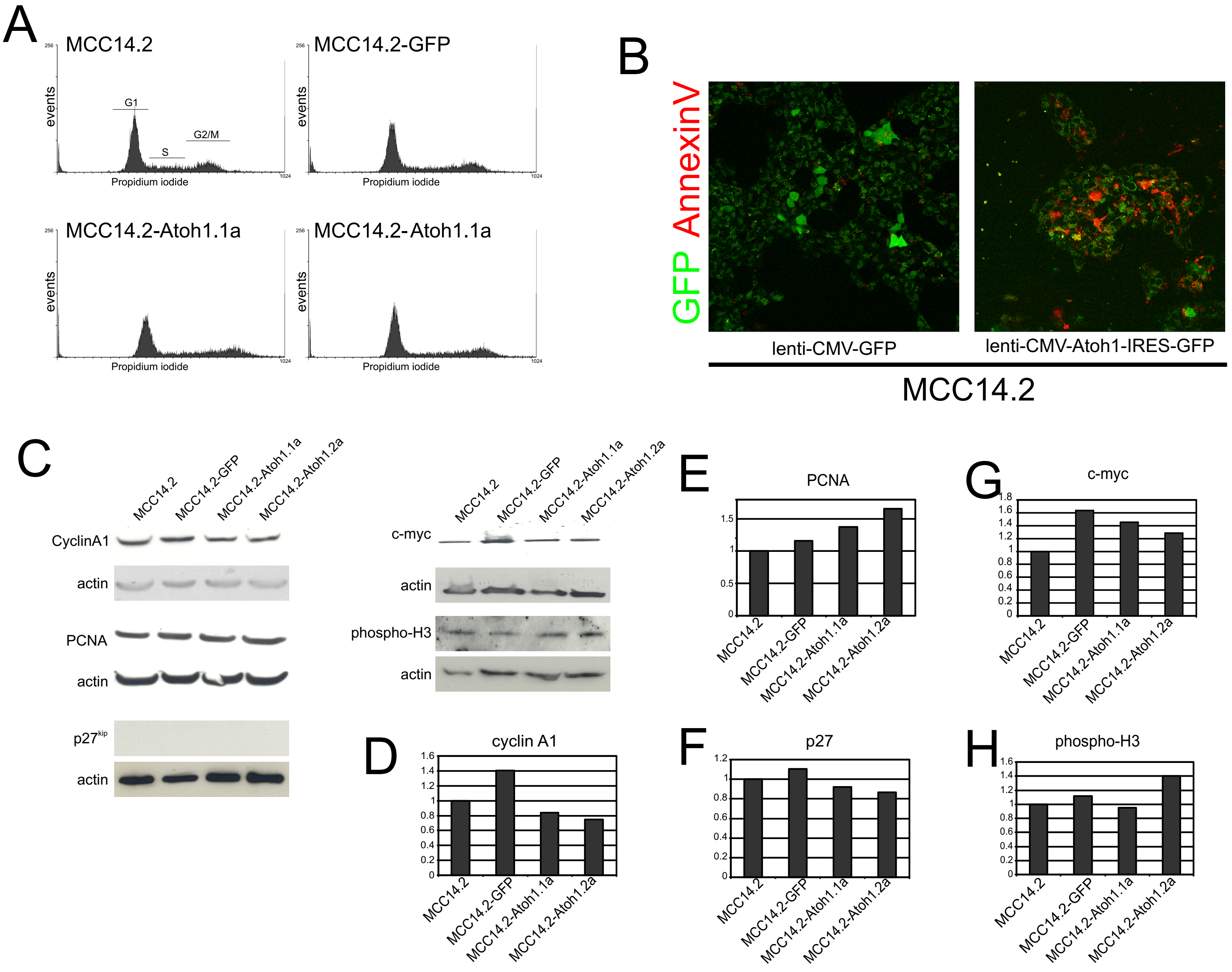

Supplementary Figure 10: A, Cell cycle distribution of MCC14.2 derived cell lines without (MCC14.2 and MCC14.2-GFP) and with *Atoh1* expression (MCC14.2-Atoh1.1a and MCC14.2-Atoh1.2a. No significant change in distribution throughout the cell cycle can be observed. **B,** Maximal projection image of AnnexinV staining (red) on cells transduced with lentiviral vectors expressing GFP (left panel) or Atoh1-IRES-GFP (right panel), GFP is in green. **C,** Western blot analysis for CyclinA1, PCNA, $p27^{kip}$, c-myc, phospho-H3 and $p21^{waf1}$ of lysates of MCC14.2 cells, MCC14.2-GFP and two MCC14.2 cell lines transduced with *Atoh1*-IRES-eGFP (MCC14.2-Atoh1.1a and MCC14.2-Atoh1.2a). The corresponding actin loading controls are shown under each blot.**D-H,** quantification of expression levels op cyclinA1 (**D**), PCNA (**E**), p27 (**F**), c-myc (**G**) and phospho-histoneH3 (**H**): representive blots are shown in panel C;
