# Supplementary material for: Atonal homolog 1 Is a Tumor Suppressor Gene
Source: PLoS Biol. 2009 Feb 24;7(2):e1000039. doi: 10.1371/journal.pbio.1000039 (PMC2652388; doi:10.1371/journal.pbio.1000039)
Supplement: Figure S11 — (A) RT-PCR for target of Notch signaling HES1 on mRNA isolated from MCC14.2 cells, MCC14.2 cells transduced with GFP, and two MCC14.2 cell lines transduced with Atoh1-IRES-eGFP (MCC14.2-Atoh1.1a and MCC14.2-Atoh1.2a). GADPH loading control is shown below. (B) Western blot analysis for cleaved intracellular Notch (NICD). Different concentrations of presinilin inhibitor X were used; a plus sign (+) indicates a positive control for cleaved NICD. (C) Different concentrations of γ-secretase inhibitor (inhibitor X) on MCC14.2 do not influence the proliferation rate. First lane: 10 μM inhibitor X, second lane: DMSO control of previous lane, third lane: 1 μM inhibitor X, fourth lane: DMSO control of previous lane, and fifth lane: untreated. (D) RT-PCR for expression of 90 tyrosine kinases scored from undetectable (white) over orange (expression) to red (strong expression) on mRNA or on untransduced MCC14.2 cells (lane 1), GFP transduced MCC14.2 cell line (lane 2), and two independent MCC14.2-derived cell lines transduced with Atoh1-IRES-eGFP vectors (lane 3: MCC14.2-Atoh1.2a) and on untransfected HT29 cells (lane14: HT29), GFP-transfected HT29 cells (lane5: HT29-GFP), and HT29 cells transfected with the Atoh1-IRES-eGFP construct (lane 6: HT29-Atoh1). (E) RT-qPCR for ATOH1 mRNA levels (compared to GADPH mRNA levels) in MCC cell lines. MCC1 (first lane: MCC cell line with endogenous high ATOH1 expression), MCC14.2 (lane 2), GFP-transduced MCC14.2 cell line (lane 3), and two independently made MCC14.2-derived cell lines transduced with Atoh1-IRES-GFP vectors (lane 4: MCC14.2-Atoh1.1a, and lane 5: MCC14.2-Atoh1.2a). (F) Western blot analysis for NTRK1 and Neurotrophin-3 (NT3) of untransduced MCC14.2 cells (lane 1), GFP-transduced MCC14.2 cell line (lane 2), and two independently made MCC14.2-derived cell lines transduced with Atoh1-IRES-GFP vectors (lane 3: MCC14.2-Atoh1.1a, and lane 4: MCC14.2-Atoh1.2a); actin loading controls are represented below each blot. (G and H) Quantif [file pbio.1000039.sg011.pdf]

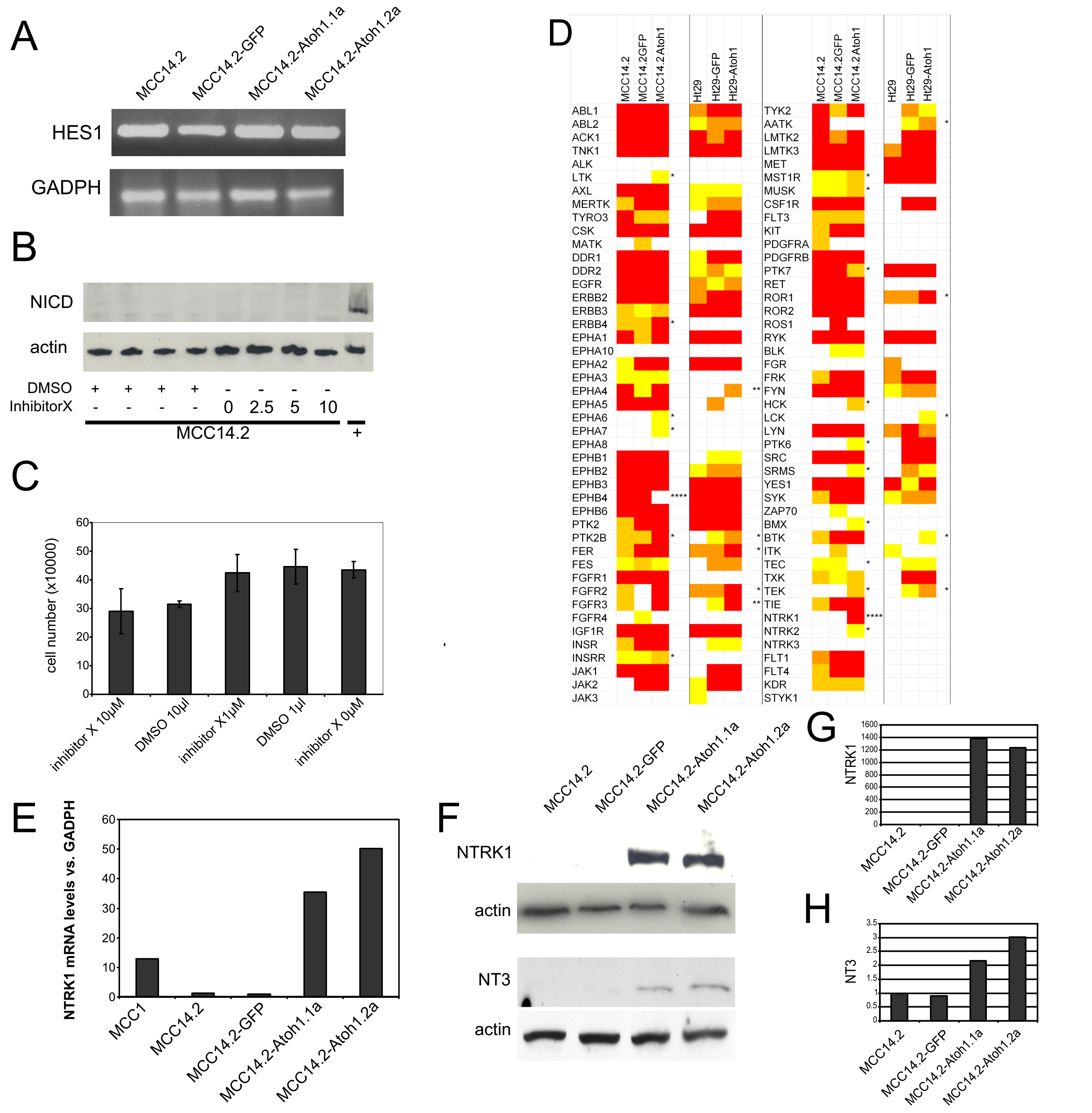

Supplemental figure 11: ATOH1 acts independently of Notch but modulates RTK exprerssion. A, RT-PCR for target of Notch signalling HES1 on mRNA isolated from MCC14.2 cells, MCC14.2 cells transduced with GFP and two MCC14.2 cell lines transduced with Atoh1-IRES-eGFP (MCC14.2-Atoh1.1a and MCC14.2-Atoh1.2a). GADPH loading control is shown below. **B,** Western blot analysis for cleaved intracellular Notch (NICD). Different concentrations of presinillin inhibitorX were used, + is a positive control for cleaved NICD. **C,** Different concentrations of gamma-secretase inhibitor (inhibitor X) on MCC14.2 do not influence the proliferation rate. First lane: 10µM inhibitor X, second lane: DMSO control of previous lane, third lane: 1µM inhibitor X, fourth lane: DMSO control of previous lane, fifth lane: untreated. **D,** RT-PCR for expression of 90 tyrosine kinases scored from undetectable (white) over orange (expression) to red (strong expression) on mRNA of on untransduced MCC14.2 cells (lane 1), GFP transduced MCC14.2 cell line (lane 2), and two independent MCC14.2 derived cell lines transduced with Atoh1-IRES-eGFP vectors (lane 3: MCC14.2-Atoh1.2a) and on untransfected HT29 cells (lane14: HT29), GFP-transfected HT29 cells (lane5: HT29-GFP) and HT29 cells transfected with Atoh1-IRES-eGFP construct (lane 6: HT29-Atoh1). **E,** RT-qPCR for *ATOH1* mRNA levels (compared to *GADPH* mRNA levels) in MCC cell lines. MCC1 (First lane; MCC cell line with endogenous high *ATOH1* expression), MCC14.2 (lane 2), GFP transduced MCC14.2 cell line (lane 3), and two independently made MCC14.2 derived cell lines transduced with Atoh1-IRES-GFP vectors (lane 4: MCC14.2-Atoh1.1a and lane 5 MCC14.2-Atoh1.2a). **F,** Western blot analysis for NTRK1 and Neurotrophin-3 (NT3) of untransduced MCC14.2 cells (lane 1), GFP transduced MCC14.2 cell line (lane 2), and two independently made MCC14.2 derived cell lines transduced with Atoh1-IRES-GFP vectors (lane 3: MCC14.2-Atoh1.1a and lane 4 MCC14.2-Atoh1.2a), , actin loading controls are represented below each blot. **G-H**, Quantifications of westerns in F, normalized to actin.
