# Supplementary material for: Atonal homolog 1 Is a Tumor Suppressor Gene
Source: PLoS Biol. 2009 Feb 24;7(2):e1000039. doi: 10.1371/journal.pbio.1000039 (PMC2652388; doi:10.1371/journal.pbio.1000039)
Supplement: Figure S12 — The quantifications are performed with USI software. The signal for the protein of interest was standardized to its respective actin loading control. Antibody is stated above each graph, representative experiments are shown in main figures: (A) Figure 4B, (B) Figure 4B, (C) Figure 4B, (D1) Figure 4C, (D1′) Figure 4C, (D2) Figure 4C, (D2′) Figure 4C, (E) Figure 5B , (F) Figure 5B, (G) Figure 6A, (H) Figure 6A, (I) Figure 6A, (J) Figure 6A, (K) Figure 6B, (L) Figure 6B, (M) Figure 6B, (N) Figure 6B, (O) Figure 6C, (P) Figure 6C, (Q) Figure 6C, (R) Figure 6E, (S) Figure 6E, (T) Figure 6E, (U) Figure 7D, (V) Figure 7D, (W) Figure 7E, (X) Figure 7E, and (Y) Figure 7E. (365 KB PDF) [file pbio.1000039.sg012.pdf]

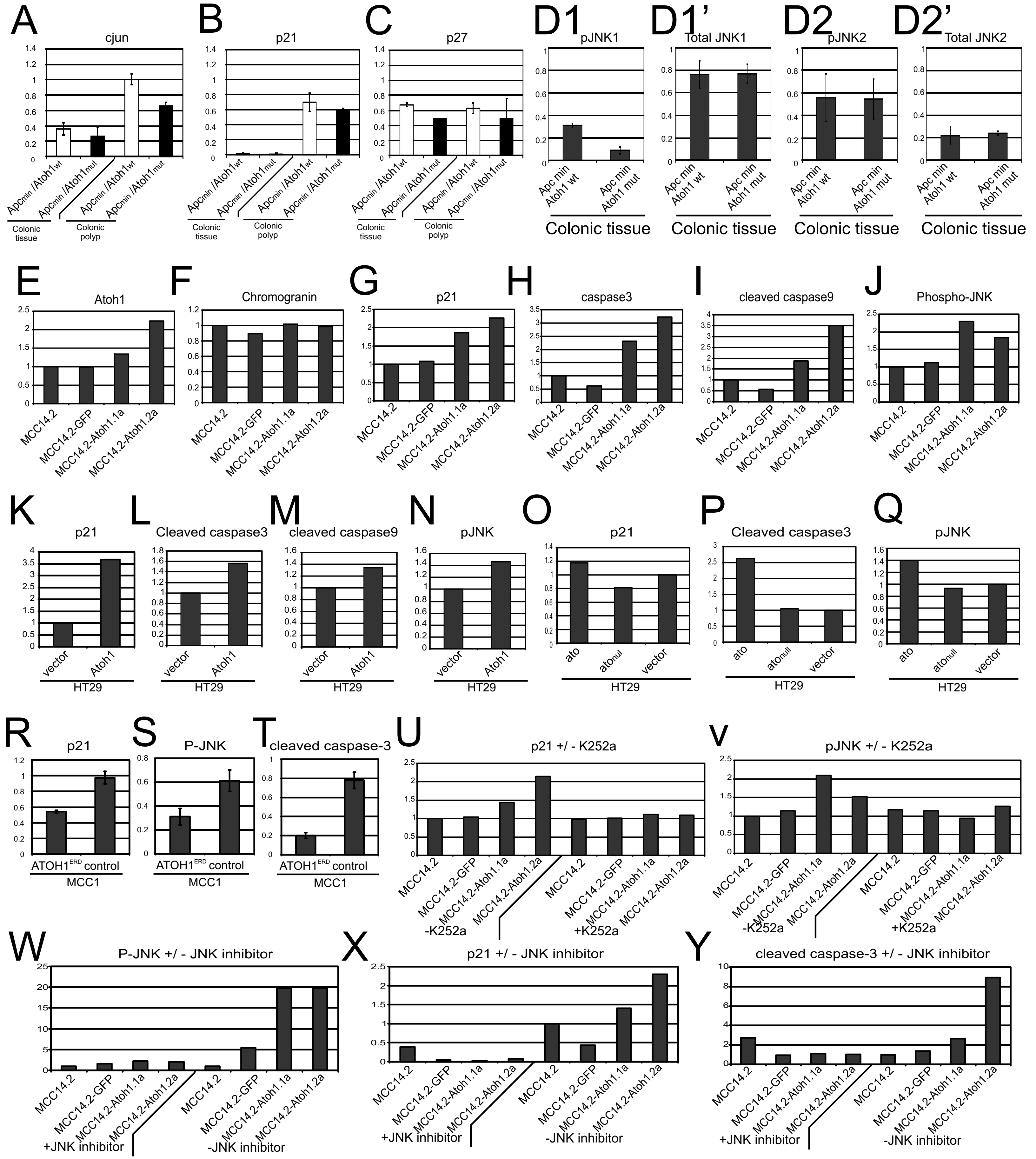

Supplementary Figure 12: Quantifications of western blot analysis in main figures (minimum of 2 blots per quantification). The quantifications are performed with USI software. The signal for the protein of interest was standardised to its respective actin loading control. Antibody is stated above each graph, representive experiments are shown in main figures (**A**) Fig4B, (**B**) Fig4B, (**C**) Fig4B, (**D1**) Fig4C, (**D1'**) Fig4C, (**D2**) Fig4C, (**D2'**) Fig4C, (**E**) Fig5B , (**F**) Fig5B, (**G**) Fig6A, (**H**) Fig6A, (**I**) Fig6A, (**J**) Fig6A, (**K**) Fig6B, (**L**) Fig6B, (**M**) Fig6B, (**N**) Fig6B, (**O**) Fig6C, (**P**) Fig6C , (**Q**) Fig6C, (**R**) Fig6E, (**S**) Fig6E, (**T**) Fig6E, (**U**) Fig7D, (**V**) Fig7D, (**W**) Fig7E, (**X**) Fig7E and (**Y**) Fig7E.
